# Detection and Typing of Plasmids in Acinetobacter baumannii Using *rep* Genes Encoding Replication Initiation Proteins

**DOI:** 10.1128/spectrum.02478-22

**Published:** 2022-12-06

**Authors:** Margaret M. C. Lam, Jonathan Koong, Kathryn E. Holt, Ruth M. Hall, Mehrad Hamidian

**Affiliations:** a Department of Infectious Diseases, Monash University, Melbourne, Australia; b Australian Institute for Microbiology and Infection, University of Technology Sydney, New South Wales, Australia; c Department of Infection Biology, London School of Hygiene and Tropical Medicine, London, United Kingdom; d School of Life and Environmental Sciences, The University of Sydney, New South Wales, Australia; The National University of Singapore and the Genome Institute of Singapore

**Keywords:** *Acinetobacter baumannii*, plasmid, replication initiation gene, Rep protein, Rep_3, Rep_1 and RepPriCT_1

## Abstract

Plasmids found in Acinetobacter species contribute to the spread of antibiotic resistance genes. They appear to be largely confined to this genus and cannot be typed with available tools and databases. Here, a method for distinguishing and typing these plasmids was developed using a curated, non-redundant set of 621 complete sequences of plasmids from Acinetobacter baumannii. Plasmids were separated into 3 groups based on the Pfam domains of the encoded replication initiation (Rep) protein and a fourth group that lack an identifiable Rep protein. The *rep* genes of each Rep-encoding group (*n* = 13 Rep_1, *n* = 107 RepPriCT_1, *n* = 351 Rep_3) were then clustered using a threshold of >95% nucleotide identity to define 80 distinct types. Five Rep_1 subgroups, designated R1_T1 to R1-T5, were identified and a sixth reported recently was added. Each R1 type corresponded to a conserved small plasmid sequence. The RepPriCT_1 plasmids fell into 5 subgroups, designated RP-T1 to RP-T5 and the Rep_3 plasmids comprised 69 distinct types (R3-T1 to R3-T69). Three R1, 2 RP and 32 R3 types are represented by only a single plasmid. Over half of the plasmids belong to the 4 most abundant types: the RP-T1 plasmids (*n* = 97), which include conjugation genes and are often associated with various acquired antibiotic resistance genes, and R3-T1, R3-T2 and R3-T3 (*n* = 95, 30 and 45, respectively). To facilitate typing and the identification of plasmids in draft genomes using this framework, we established the Acinetobacter Typing database containing representative nucleotide and protein sequences of the type markers (https://github.com/MehradHamidian/AcinetobacterPlasmidTyping).

**IMPORTANCE** Though they contribute to the dissemination of genes that confer resistance to clinically important carbapenem and aminoglycoside antibiotics used to treat life-threatening Acinetobacter baumannii infections, plasmids found in Acinetobacter species have not been well studied. As these plasmids do not resemble those found in other Gram-negative pathogens, available typing systems are unsuitable. The plasmid typing system developed for A. baumannii plasmids with an identifiable *rep* gene will facilitate the classification and tracking of sequenced plasmids. It will also enable the detection of plasmid-derived contigs present in draft genomes that are widely ignored currently. Hence, it will assist in the tracking of resistance genes and other genes that affect survival in the environment, as they spread through the population. As identical or similar plasmids have been found in other Acinetobacter species, the typing system will also be broadly applicable in identifying plasmids in other members of the genus.

The plasmids found in Acinetobacter species clearly differ from the better studied and understood plasmids found in the majority of Gram-negative species and covered by the PlasmidFinder database (1). Indeed, the plasmids found in other Gram-negative species (especially Enterobacterales) do not appear to be stably maintained in Acinetobacter species and Acinetobacter plasmids are not seen in other Gram-negative pathogens. Hence, a typing and classification scheme relevant to Acinetobacter plasmids is needed.

In 2010, sequences were available for very few Acinetobacter plasmids, and they were mainly derived from the modest number of complete genomes of A. baumannii isolates that had been published at that time. At this point, an analysis aimed at generating a PCR detection and typing scheme based on *rep* genes encoding replication initiation (Rep) proteins was published (2). Fifteen complete plasmid sequences, 12 of them derived from 5 available complete genomes, together with 8 partial sequences (5 determined in the study) were analyzed. Four plasmids were identical or had identical or very closely related *rep* genes, and 2 plasmids included 2, and another has 3 *rep* genes. Hence, a total of 24 different *rep* gene sequences were examined. The majority encoded Rep proteins belonging to Pfam01051 corresponding to the Rep_3 family. The Rep proteins of 2 plasmids belonged to the Rep_1 family (Pfam01446), and 1 plasmid encoded a protein with a Rep motif (Pfam03090). As the method developed used PCR to detect the *rep* genes, a cutoff 74% nucleotide identity in the *rep* gene sequence was used to group the *rep* genes and ensure specificity of the primers. Nineteen groups (GR1-GR19) were proposed. However, some of these groups encompassed two clearly distinct *rep* gene types and a secondary classification assigned Aci numbers up to 10 to some of the Rep proteins, e.g., the replicases associated with the 2 distinct groups in GR2 were designated Aci1 and Aci2 (2).

Using the PCR approach, an analysis of 96 multiply antibiotic resistant isolates, mainly from Europe, by the same group found that *rep*Aci1 (GR2) and *rep*Aci6 (GR6) were the most widely distributed (3). However, as the cost of genome sequencing fell, over the next few years many more complete or draft genomes became available and PCR typing was never extensively used. Consequently, such broad groups including members with *rep* genes that differ by up to 26% nucleotide identity are no longer necessary or practical. Moreover, in the absence of either a centralized database or a clear revision of the rules for grouping, assignment of additional groups has been problematic. For example, the GR20 designation has been used for at least 3 different types (4–6). In other cases, examples of new types have been identified but GR numbers were not assigned (e.g (7, 8).

In 2017, a review examined only the small (< 10 kbp) Acinetobacter plasmids and, based on a phylogeny of the Rep_3 proteins, separated plasmids encoding the distinct Aci1 and Aci2 types of the GR2 group with the Aci2 type becoming GR20 (5). Though this separation breaches the 74% rule as these *rep* genes are close to 80% identical, it appears to have prevailed in more recent studies (see below). However, the distinct subtypes found in other GR were not separated and additional groups were not identified. The number of groups was expanded to 23 in a 2017 study that compared 3 plasmids from a single A. baumannii isolate from Argentina to a database of 122 Rep proteins found in A. baumannii plasmids (9). A cutoff 85% protein identity was used to split GR8 into 2 groups, GR8 and GR23, and 2 further GR (GR21 and GR22) were reported (9). In 2020, a comprehensive analysis of complete plasmids from A. baumannii available in 2016 added several further GR up to GR33 using a cutoff > 74% nucleotide identity (10). Finally, in 2021, GR34 was assigned, again based on the original cutoff > 74% DNA identity (11).

A shortcoming of the GR scheme for *rep* genes arises from the fact that a number of plasmid types found in A. baumannii and in other Acinetobacter species do not include an identifiable *rep* gene and some of these are common (12–14). To overcome this, a few further studies have attempted to address the issue of Acinetobacter plasmid classification using various different data sets (new sequences and published sequences) and different approaches. Salto and coworkers (15) looked at replication, mobilization and conjugative transfer genes in a group of plasmids from various Acinetobacter species. They also identified 16 groups of Rep_3 proteins that were designated AR3G1–AR3G16 from plasmids recovered from various species but the numbering of these AR3 groups does not correspond to the group numbers used by Bertini et al. (2) and a key was not provided. In 2020, Mindlin and coworkers (14) published a study of plasmids isolated from Acinetobacter lwoffii isolates recovered from permafrost, where they used a classification system for plasmids from any Acinetobacter species based on size, then whether a *rep* or a *mob* gene, both or neither was present. They used the Salto et al. groupings for the Rep_3 proteins and a cutoff 95% protein identity for clustering.

The study of Salgardo Camargo and coworkers (10) examined 18 new sequences and 145 A. baumannii plasmids for which complete sequences were available in 2016 and grouped them using an approach that attempted to classify the whole plasmids into lineages without accounting for the many known accessory regions that should be removed to reveal the plasmid backbone. Hence, their approach is confounded by the extent of the variation caused by the acquisition and loss of significant portions of plasmids with closely related backbones, as occurs for example in the small to medium sized Rep_3 plasmids that carry acquired *dif* modules where the size of the *dif* module and other accessory content can exceed that of the backbone (e.g., (7, 16–18). They retained the > 74% nucleotide identity in *rep* genes cutoff used by Bertini and coworkers (2) and removed GR23 but kept GR20. They added 10 GR bringing the total to 33 GR. Nine of the additional plasmid types encoded Rep, Rep_1 and Rep_3 replication initiation proteins; the tenth, represented by a single plasmid, encoded a protein with a RepC motif that is not involved in replication of the plasmid (see below). A phylogeny of the Rep_3 proteins revealed distinct subgroups within some GR, as was found previously (2).

Here, we have analyzed the plasmids from A. baumannii with a complete sequence available in the GenBank nucleotide database as of February 2021 in order to develop a unified system that will allow plasmids to be typed simply and rapidly and to facilitate identification of plasmids in draft genome sequences. To reduce the plasmid data set to a manageable size, only A. baumannii plasmids were included as these are the most important from a clinical perspective and, as a first step, only those encoding a Rep protein have been typed. The most appropriate criteria for defining plasmid groups based on *rep* genes was re-assessed in the light of the fact that PCR is no longer the main source of information about the types of plasmids carried by A. baumannii isolates. A new typing and numbering system was developed but for ease of comparison, the earlier GR designations are indicated, where relevant. An online resource that includes a database of representative *rep* gene and Rep protein sequences was developed and has been made available via GitHub.

## RESULTS

### Curation of the plasmid database.

All complete sequences for A. baumannii associated plasmids were downloaded (February 2021) and, after removal of redundancies, further curation removed additional plasmids from the data set (see Materials and Methods and reasons listed in Table S1). Of particular note, sequences that were previously classified as lineage 10 (10) and assigned to GR3 were removed as they have been shown to represent a circular form of the AbGRI3 resistance island that has failed to assemble to the correct location on the chromosome (19). The segment includes only an incomplete and inactive *rep* gene (see [20] for details). The final data set comprised 621 plasmids, 539 of them derived from genome sequencing projects. A small number of partial sequences reported by Bertini et al. (2) were also included in the analysis to facilitate correlation with the typing scheme devised by those authors but were not included in plasmid counts.

### Detection of *rep* genes.

A comprehensive approach to detection of *rep* genes found in the plasmids in the data set (see Materials and Methods) included curation to remove genes incorrectly annotated as *rep* genes (described in more detail below). A total of 142 plasmids (23%) did not encode an identifiable Rep protein. Many of the plasmids in this group were related to known plasmids, including *n* = 27, 7 and 20 identical to the well characterized, small plasmids pRAY* (13), pD36-1 (17), pA85-1b (21). Thirty one had backbones related to the backbones of large conjugative plasmids where a *rep* gene of a novel, as yet unidentified, type may be present. Among these, *n* = 20 were related to pAB3 and pA297-3 (12), 8 related to pNDMBJ01 (22) and 3 related to pALWED1.1 (23). Investigation of the RepC protein encoded only by the plasmid pAB3 that was used to define GR33 (10), revealed that it is not found in known relatives with closely related backbones such as pA297-3 (12) and it was traced to the genomic island GI*sul2* (24) which is found in pAB3 but not in its close relatives. As the related plasmids do not include an identifiable *rep* gene, pAB3 was included in the no *rep* plasmid group. This highlights the importance of careful curation to identify problems that arise when bioinformatic approaches are used without reference to underlying knowledge of which regions are plasmid backbone and which are accessory. The remaining (*n* = 46) plasmids with no identifiable *rep* gene were not further examined in this study.

At least one *rep* gene was identified in 479 plasmids (77%) (Table 1). However, 19 plasmids included 2 *rep* genes and 2 included 3 *rep* (Table 1), yielding in total 502 *rep* genes. The product of each *rep* gene was screened for Pfam domains associated with replication initiation (Rep) proteins (see Materials and Methods).

**TABLE 1:** Summary of plasmid sequence data studied

Plasmids	No.
Complete plasmids	621
Part of Genomes	539
Not part of a genome project	82
No Rep	142
With Rep^*a*^	479
Plasmids encoding Rep_3	359
Plasmids encoding Rep_1	13
Plasmids encoding RepPriCT_1	107
Total plasmids	479

a 457, 19, and 2 plasmids encode 1, 2, and 3 Reps, respectively.

### Classification of plasmids carrying *rep* genes.

To simplify classification, we have used the Pfam of the replication initiation protein encoded by each *rep* gene for initial grouping of the plasmids, as this can be obtained readily. Plasmids encoding a Rep_1 family (Pfam01446) replicase were least abundant with only 13 plasmids in this group (Table 1). The Rep_3 (Pfam01051) group predominated with 359 plasmids and all the plasmids carrying multiple *rep* genes fell into this category. The Rep (Pfam03090) group that encode replicases that also include a PriCT_1 (Pfam08708) motif, here referred to as the RepPriCT_1 group, included 107 plasmids. The distinct types within each Pfam group were then defined by clustering the *rep* nucleotide sequences (see Materials and Methods), using a cutoff 95% nucleotide identity as this appeared to best separate the clearly distinct types without recording minor variations in DNA sequence. However, for most types identified using this approach, all represented *rep* genes were > 99% identical.

A total of 80 types were identified using this approach: 6 Rep_1 types, 69 Rep_3 types and 5 RepPriCT_1 types. The plasmid types identified in this way have been prefaced R1, R3, and RP for the Rep_1, Rep_3 and RepPriCT_1 groups, respectively, followed by an assigned number, generally in the order of identification or the relative abundance of the type. To facilitate comparison to earlier studies, where relevant the GR number is indicated in Tables.

### The small Rep_1 plasmids.

In the original plasmid classification (2), one plasmid, p4ABAYE (GR14) and a partial sequence from pAB49 (GR16) encoded a Rep protein belonging to the Rep_1 family (Pfam01446). The complete sequence of pAB49 is now available (GenBank accession number L77992.1) and, though it was not detected in our initial search for complete plasmids, was added to the complete plasmid database.

Only 11 additional Rep_1 plasmids were found among the 621 plasmids examined here. Of these, the complete sequences of 7 were identical to either p4ABAYE or pAB49 and these groups were designated R1-T1 and R1-T2, respectively (Table 2). Within each type, very little variation in the sequences was observed indicating that these plasmids are well conserved. These types were also widely distributed as they were recovered in various countries (Table 2). Three additional types (R1-T3 to R1-T5), each represented by only 1 or 2 examples, were detected. An additional Rep_1 plasmid type was reported recently (25) and has been added to Table 2 as R1-T6.

**TABLE 2:** Properties of plasmids encoding Rep_1 (Pfam01446) family replication initiation protein

Rep Type	Plasmid name	Length (bp)	Strain name	Yr	Country	GenBank acc. no.	Rep protein id
R1-T1	p4ABAYE^*a*^	2,726	AYE	2001	France	CU459139.1	CAM84626.1
R1-T1	pA85-1	2,726	A85	2003	Australia	CP021783.1	AHM95258.1
R1-T1	pMRSN58-2.7	2,725	MRSN 58	2010	USA	CM003316.1	KLT88566.1
R1-T1	p4ZQ6	2,725	ZQ6	2016	Iraq	CM009041.2	PQL72120.1
R1-T1	p4ZQ5	2,725	ZQ5	2016	Iraq	CM009683.1	PQL83905.1
R1-T2	pAB49^*b*^	2,343	Ab49	Nk^*c*^	Nk	L77992.1	AAA99423.1
R1-T2	pA85-1a	2,343	A85	2003	Australia	CP021784.1	ASF79212.1
R1-T2	pMRSN7339-2.3	2,343	MRSN 7339	2004	USA	CM003313.1	KLT74217.1
R1-T2	pDA33382-2	2,359^*d*^	DA33382	Nk	Germany	CP030108.1	AXB17575.1
R1-T3	pAb-D10a-a_5	2,697	Ab-D10a-a	2016	Ghana	CP051874.1	QJF33731.1
R1-T3	pAb-B004d-c_4	2,697	Ab-B004d-c	2016	Ghana	CP051879.1	QJF37612.1
R1-T4	p3AB5075	1,967	AB5075-UW	2008	USA	CP008709.1	AKA33693.1
R1-T5	pTS236	2,252	DS002	Nk	Nk	JN872565.1	AFB69861.1
R1-T6	pMRSN56-1	2,178	MRSN56	2010	USA	CP080453.1	QYM41639.1

a GR14 in (2).

b GR16 sequence was reported as partial in (2) but completed later in 2016.

c Not known.

d Length difference is likely due to an assembly issue.

**FIG 1:**
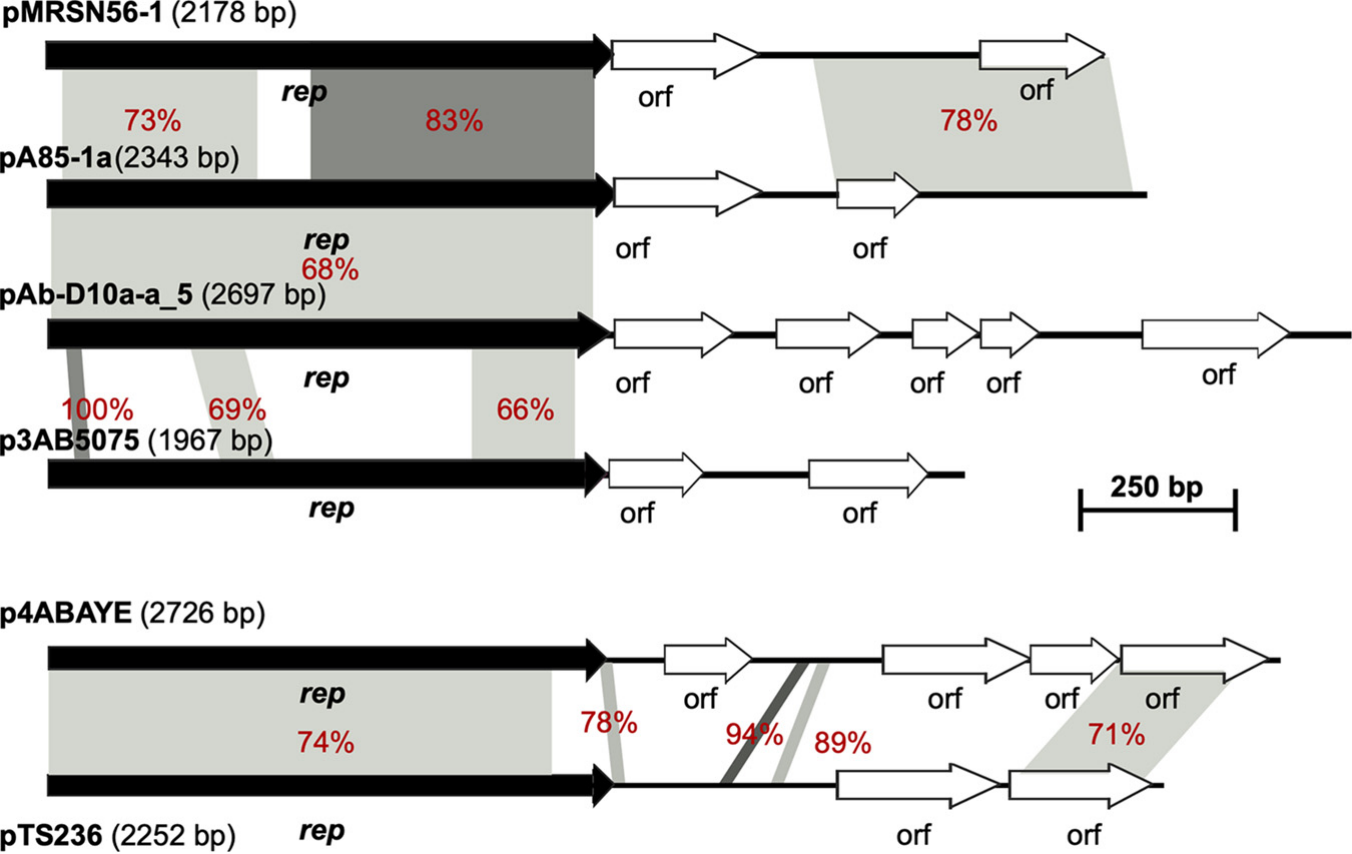
Comparison of representatives of plasmids encoding Rep_1 family (Pfam01446) Rep proteins. Black horizontal arrows show indicate *rep* genes. White arrows encode hypothetical proteins. Regions with significant DNA identities are shown using shades of gray with % identities also labeled in red. p4ABAYE (GenBank accession number CU459139), pA85-1a (GenBank accession number CP021784), pAb-D10a-a_5 (GenBank accession number CP051874), p3AB5075 (GenBank accession number CP008709), pTS236 (GenBank accession number JN872565), and pMRSN56-1 (GenBank accession number CP080453) represent R1-T1, R1-T2, R1-T3, R1-T4, R1-T5, and R1-T6, respectively.

Plasmids with a Rep_1 replication initiation protein are generally small and replicate using a rolling circle mechanism. The Rep_1 plasmids identified here were all less than 3 kbp in length and do not include any antibiotic resistance genes. Most were found in completed genome sequences. However, due to their small size, they may be missed in genomes derived using long read sequencing (26). The relationship between the sequences of the Rep proteins encoded by each type is shown in Table 3. There appear to be 2 distinct groups based on alignments with significant levels of identity and > 75% coverage, namely T1 and T5 in one group and T2, T3, T4, and T6 in the other. The structures of one plasmid of each type in the R1 group are shown in Fig. 1.

**TABLE 3:** Pairwise protein identities of representative Rep_1 type Rep proteins^*a*^

Type	P1-T1	P1-T2	P1-T3	P1-T4	P1-T5	P1-T6
P1-T1	100	25.2 (27)	28.2 (31)	-^*b*^	73.4 (82)	26.68 (36)
P1-T2		100	63.7 (89)	44.4 (82)	22.7 (28)	73.28 (100)
P1-T3			100	45.2 (76)	26.7 (30)	65.63 (90)
P1-T4				100	-	44.58 (82)
P1-T5					100	24.8 (28)
P1-T6						100

a Numbers in bracket indicate % coverage.

b No significant matches.

### The RepPriCT_1 family plasmids.

In the original classification, a single plasmid pACICU2, carried a gene encoding a Rep protein belonging to the Rep and PriCT_1 families. The plasmid type was GR6 and the Rep protein was designated Aci6 (2). Plasmids of this type have been implicated in the dissemination of the *oxa23* gene (carbapenem resistance) and the *aphA6* gene (amikacin resistance). They include a complete set of genes for conjugation, and some have been shown to be conjugative (27–29). Among the 107 complete RepPriCT_1 plasmids analyzed here, the majority (*n* = 97) were *rep*Aci6 plasmids, here designated RP-T1. However only 10 representatives are listed in Table 4 selected to include well characterized examples and to illustrate the global distribution of plasmids of this type. A complete list can be found in Table S2. Where it has been examined, these plasmids share a, though not completely identical, backbone that is often interrupted by transposons encoding either antibiotic resistance genes or heavy metal resistance genes (28–31). Hence, they are one of the most important plasmid types implicated in introducing further antibiotic resistance genes into A. baumannii isolates.

**TABLE 4:** Properties of plasmids encoding RepPriCT_1 family (Pfam03090) replication initiation protein

Rep type^*a*^	Plasmid		Date	Country	Strain name	Accession no.	Rep protein id
	Name	Length (bp)					
RP-T1	pACICU2^*b*^	70,101	2005	Italy	ACICU	CP031382	QCS04021
RP-T1	pAb-G7-2^*b*^	70,100	2002	Australia	G7	KF669606	AGY56182
RP-T1	pA85-3^*b*^	86,334	2003	Australia	A85	CP021787	ASF79226
RP-T1	pS21-2^*b*^	123,432	1996	Singapore	SGH9601	MG954377	AWO68424
RP-T1	pMC23.1	67,441	2016	Bolivia	MC23	MK531538	QCO89676
RP-T1	pCS01A^*c*^	63,720	< 2014	Switzerland	CS01	HG977523	-^*c*^
RP-T1	pTG22182_1	71,233	2011	USA	TG22182	CP039994	QCO84474
RP-T1	p3VB958	82,500	2019	India	VB958	CP040043	QCP18569
RP-T1	pAB04-2	87,569	2012	Canada	Ab04-mff	CP012008.1	AKQ32652.1
RP-T1	p2K50	79,598	2008	Kuwait	K50	LT984690	SPC58401
RP-T2	pABTJ1^*d*^	77,528	Nk^*e*^	China	MDR-TJ	CP003501	AFI97405
RP-T2	pAZJ221^*f*^	77,530	2009	China	A221	KM922672	AJF79875
RP-T2	p1BJAB07104	70,170	2007–8	China	BJAB07104	CP003887	AGQ16162
RP-T2	p2BJAB0868	70,167	2007–8	China	BJAB0868	CP003888	AGQ12301
RP-T2	pHRAB-85	77,513	2014	China	HRAB-85	CP018144	APF45706
RP-T2	pBJ83	69,069	2007	China	XDR-BJ83	CP018422	APM50974
RP-T2	pABAY10001_1C	54,627	< 2019	South Korea	ABAY10001	MK386682	QBN23313
RP-T3	pKBN10P02143^*g*^	52,517	2012	South Korea	KBN10P02143	CP013925	ALY01450
RP-T4	p2ZQ6	6,772	2016	Iraq	ZQ6	CM009039	PQL72098
RP-T5	p4.36-1512	4,721	2016	Russia	36-1512	CP059390	QLY88352
RP-T5	pE47_007	4,715	2013	Australia	E47	CP042563	QFH47718

a 94 total plasmids including pAbG7-2, pA85-3, pD46-3, pS32-2 have a Rep protein greater than 99% identical to Rep encoded by pACICU2. Ten representatives are shown, and a complete list is in Supplementary Table S2. This type corresponds to GR6.

b Shown to conjugate (16, 27–29).

c A second plasmid pCS01B is identical to pCS01A but the *rep* gene in incomplete. Entry does not include annotations and hence no protein id.

d pABTJ1 is grouped as GR25 in (10).

e Not known.

f Shown to be conjugative (6). As for pABTJ1, it carries Tn*2009* (containing *oxa23*).

g pKBN10P02143 is designated as GR32 (10).

A group of 7 plasmids were close relatives of pABTJ1 (32) and this type, here designated RP-T2, corresponds to GR25. The backbone of plasmid pABTJ1 has been shown to include a set of genes that encode proteins involved in conjugation that are related to those encoded by RP-T1 (Aci6) plasmids (33). Another plasmid of this type also carrying the carbapenem resistance transposon Tn*2009* has been shown to be conjugative (6). These plasmids are so far confined to Asia (Table 4). Three further types, RP-T3 to RP-T5 were each detected in only 1 or 2 plasmids (Table 4) with RP-T3 corresponding to GR32, and no reports describing them were found.

The relationships between the Rep proteins encoded by the types in this group are shown in Table 5. This comparison revealed 2 broad subgroups, with RP-T1 Rep grouping with RP-T2, and RP-T4 grouping with RP-T5 while RP-T3 was more distantly related to the other types. RP-T4 and RP-T5 plasmids are substantially smaller than the conjugative RP-T1 and RP-T2 plasmids.

**TABLE 5:** Pairwise protein identities of RepPriCT_1 group representatives^*a*^

Rep type	Plasmid	Protein id	pACICU2	pABTJ1	pKBN10P02143	p2ZQ6	p4.36-1512
RP-T1	pACICU2	QCS04021.1	100	69.23 (98)	38.7 (95)	38.6 (83)	37.1 (85)
RP-T2	pABTJ1	AFI97405.1		100	39.7 (89)	40.8 (79)	36.6 (80)
RP-T3	pKBN10P02143	ALY01450.1			100	35.9 (98)	35.2 (71)
RP-T4	p2ZQ6	PQL72098.1				100	69.3 (100)
RP-T5	p4.36-1512	QLY88352.1					100

a Numbers in bracket indicate % coverage.

### Identification of the *rep* gene in Rep_3 plasmids.

**FIG 2:**
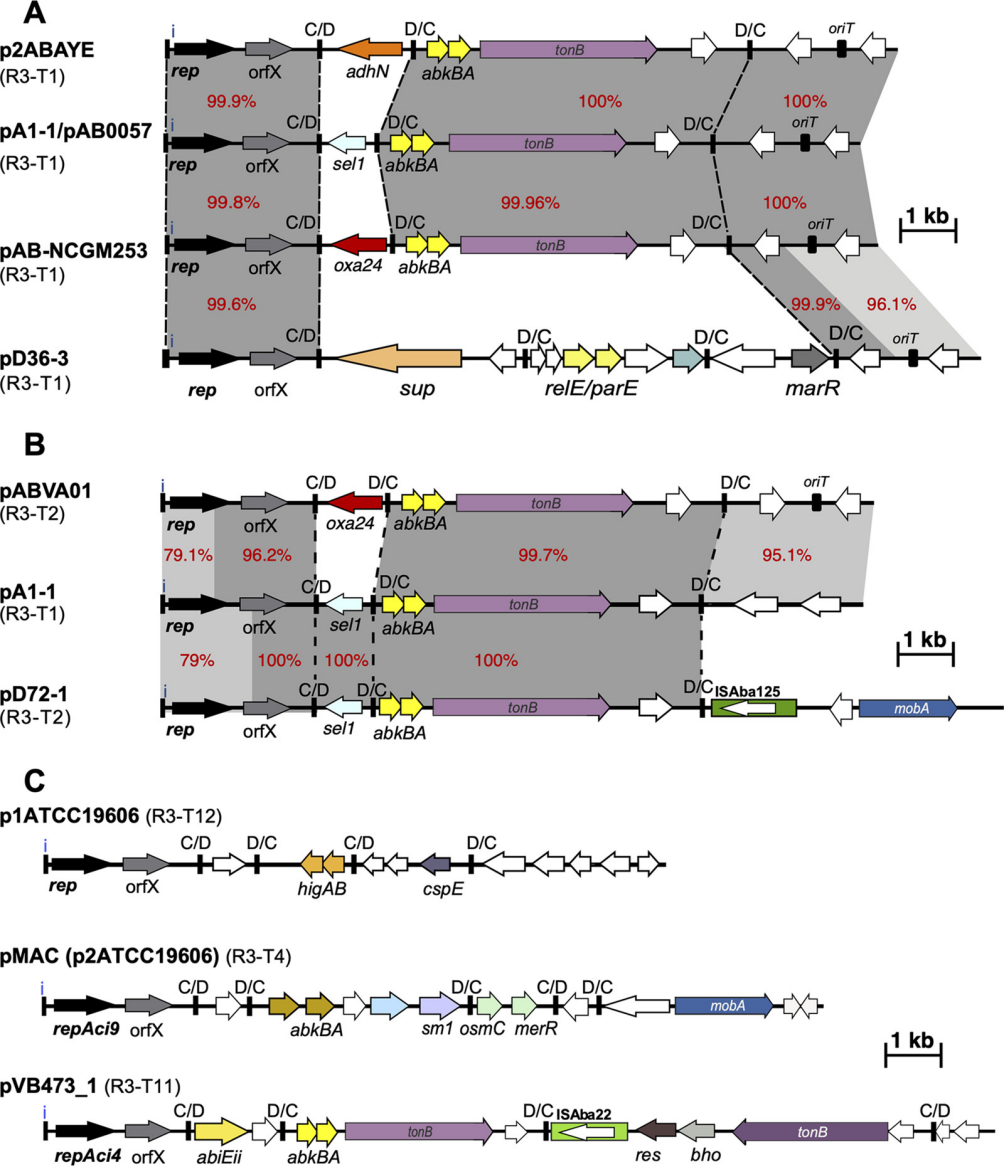
Schematic comparison of Rep_3 family (Pfam01051) plasmid structures. Horizontal arrows show the length and orientation of genes with *rep* genes colored black, resistance genes red, toxin/anti-toxins yellow and mobilization genes blue. Green boxes indicate insertion sequences with their transposase shown inside the box. Small thick vertical bar marked with “i” indicate iterons. Other vertical bards marked with “C/D or D/C” indicate the location of p*dif* sites. Regions with significant DNA identities are shown using shades of gray with % identities also shown using red numbers. Dotted lines draw the show the boundaries of p*dif* modules. (A) represents variations within five R3-T1 plasmids (p2ABAYE, pA1-1/pAB0055, pAB-NCGM253, and pD36-3 with GenBank accession numbers CU459138, CP010782/CP001183, AB823544, and CP012955, respectively) that carry different p*dif* modules. (B) compares two R3-T1 plasmids pABVA01 and pD72-1 (GenBank accession numbers FM210331.1 and KM051986, respectively) with pA1-1 (GenBank accession number CP010782) representing R3-T1. (C) represents the structure of plasmids representing R3-T12 (p1ATCC19606; GenBank accession number CP045108), R3-T4 (pMAC; AY541809) and R3-T11 (pVB473_1; GenBank accession number CP050389) with no significant DNA identity.

There has been significant confusion in the literature and in the annotations of plasmid sequences in GenBank with respect to the identification and naming of the *rep* gene in many of the plasmids in this category. The confusion applies particularly to plasmids with the configuration shown in Fig. 2, and complicated the identification of *rep* genes for inclusion in the *rep* gene database. The confusion appears to have arisen early when the *rep* gene was designated *repB* and the adjacent downstream gene, which we have labeled orfX in Fig. 2 as its function is currently unknown, was called *repA* (34). In fact, a published study had previously demonstrated that only a single gene (the one designated *repB*) and upstream iterons are essential for replication of pMAC (p2ATCC19606), and the *rep* gene, which encodes the Aci9 replicase (2), was designated *repM* (35). The presence of orfX, which encodes a helix-turn-helix domain protein, in the plasmid set used by Bertini and coworkers (2) is also shown in Table 6. Currently, the downstream orfX gene continues to be misidentified in many publications and GenBank entries as the *rep* gene, and manual curation (see Materials and Methods) was used to ensure that these genes were not included in our databases.

**TABLE 6:** Properties of plasmids encoding Rep_3 family proteins described in Bertini et al., 2010

Plasmid	Rep	GR group	Length (aa)	Iterons	OrfX	C/D	Protein_id	Plasmid length (bp)	Strain	Accession no.	No. complete 95% (99%)
pAB0057	Aci1	2	316	4	Y	Y	ACJ43223.1	8,731	AB0057	CP001183	95 (88)
p2ABAYE	Aci1	2	316	4	Y	Y	CAM84615.1	9,661	AYE	CU459138	95 (88)
pACICU1	Aci1	2	316	4	Y	Y	ACC58980.1	28,279	ACICU	CP000864	95 (88)
pAB2	Aci1	2	316	4	Y	Y	ACC58980.1	11,302	ATCC17978	CP000523	95 (88)
pABVA01	Aci2	2	316	4	Y	Y	CAR65318.1	8,963	VA-566/00	FM210331	30 (29)
p203	Aci3	3	307	4	Y^*a*^	Y^*a*^	ADM89092.1	>1,068	Ab203	GU978997	13 (13)
p844	Aci4	4	307	4	Y^*b*^	Y^*b*^	ADM89093.1	>1,119	Ab844	GU978998	8 (8)
p537	Aci5	5	310	4	Y^*c*^	Y^*c*^	ADM89094.1	>1,125	Ab537	GU978999	2 (0)
pAb736	Aci7	3	306	4	Y^*d*^	Y^*d*^	ADM89091.1	>1,065	Ab736	GU978996	1 (0)
p11921	Aci8	8	307	4	N^*e*^	N^*e*^	ADM89095.1	>1,103	Ab11921	GU979000	2 (2)
pMAC	Aci9	8	311	4	Y	Y	AAT09649.1	9,540	ATCC19606	AY541809	15 (13)
pACICU1	Aci10 (AciX)	10	315	4	N	N	ACC58984.1	28,279	ACICU	CP000864	4 (4)
p1ABAYE	p1ABAYE0001	11	316	4	Y	Y	CAM84608.1	5,644	AYE	CU459137	4 (4)
pABIR	RepA_AB	12	295	4^*f*^	Y	Y	ACB05788.1	29,823	Ab1	EU294228	10 (1)
pAB02	Rep_AB	12	295	4	Y	Y	AAR00517.1	>4,162	Ab02	AY228470	10 (9)
p3ABAYE	p3ABAYE0002	13	390	-	N	N	CAM84695.1	94,413	AYE	CU459140	10 (7)
p1ABSDF	p1ABSDF0001	1	314	4	N	N	CAP02936.1	6,106	SDF	CU468231	1 (1)
p2ABSDF	p2ABSDF0001	12	297	4^*f*^	Y	Y	CAP02944.1	25,014	SDF	CU468232	8 (8)
p2ABSDF	p2ABSDF0025	18	339	4	N	N	CAP02966.1	25,014	SDF	CU468232	1 (1)
p3ABSDF	p3ABSDF0018	15	303	4 ^*f*^	Y	Y	CAP02992.1	24,922	SDF	CU468233	1 (1)
p3ABSDF	p3ABSDF0002	7	310	-	N	N	CAP02976.1	24,922	SDF	CU468233	1 (1)
p3ABSDF	p3ABSDF0009	9	326	4^*f*^	N	N	CAP02983.1	24,922	SDF	CU468233	1 (1)
pAB1	A1S_3471	17	318	4	Y	Y	ABO13860.1	13,408	ATCC17978	CP000522	1 (1)
p135040	135040	19	318	4	nk	nk	ACX70400.1	>3,975	135040	GQ861437	0 (0)

a p203 is incomplete however another plasmid with an identical RepAci3 (pD1279779; GenBank no. CP003968) includes orfX and C/D sites downstream of the Rep.

b p844 is incomplete, (the RepAci4 plasmid pPM194122_2; GenBank no. CP050427) includes orfX and C/D sites downstream of the Rep.

c p537 is incomplete but plasmid II of the strain R2091 (GenBank no. LN997847) with 98.6% DNA identity (compared to *repAci5* of p537) was analyzed.

d pAb736 is incomplete however, the closet plasmid is pRCH52-1 (GenBank no. KT346360), *repAci7* and is 95% DNA identical to that of pRCH52-1.

e Given that p11921 is incomplete the presence of orfX and its downstream C/D sites were investigated in pAb825_36 (GenBank no. MG100202), which encodes a Rep identical to p11921. pAb825_36 has iteron sequences upstream of its two *rep* genes.

f Imperfect iterons (1 to 3 bp differences).

### Overview of the Rep_3 group.

Rep_3 plasmids were the most abundant in this collection and constitute the most diverse group. Classification of the 382 *rep* genes encoding Rep_3 replication initiation proteins using the cutoff > 95% nucleotide identity for the *rep* gene revealed 69 distinct types (Table 7). A complete list of the members of each type is found in Table S3. However, only 8 types (T1-T8) included 10 or more members (accounting for 63% of *rep* genes encoding a Rep_3 type protein) and the majority of types are represented by only 1 to 4 members. When only representatives in the Bertini *rep* gene set are considered but a 95% nucleotide identity cutoff is used more than half of the *rep* genes in the larger Rep_3 plasmid set could be classified (last column in Table 6). This is largely due to the predominance of R3-T1 (95 of 382 total) and R3-T2 (30 of 382) plasmids both of which were originally included in GR2 and encode, respectively, an Aci1 or Aci2 replication initiation protein. However, in the larger analysis, the R3-T3 (GR24) group, which is exemplified by pABTJ2 (36) is also abundant, including 45 members.

**TABLE 7:** Rep_3 family types

Type Rep	Plasmid name	Aci	No. 95% (99%)	Group^*b*^	Protein id	Accession no.
R3-T1	pAB0057^*a*^ (pA1-1)^*c*^	Aci1	95 (88)	GR2	ACJ43223.1	CP001183.2
R3-T2	pD72-1 (pABV01^*a*^)^*d*^	Aci2	30 (29)	GR2/20	AIH07953.1	KM051986.1
R3-T3	pABTJ2	-	45 (44)	GR24	AGG91013.1	CP004359.1
R3-T4	pMAC^*a*^ (p2ATCC19606)	Aci9	15 (13)	GR8	AAT09649.1	AY541809.1
R3-T5	pABLAC1	Aci3	13 (13)	GR3	AIY39145.1	CP007713.1
R3-T6	p1ZQ9	-	13 (13)	-	PQJ03469.1	CM009083.3
R3-T7	p3ABAYE^*a*^	-	10 (7)	GR13	CAM84695.1	CU459140.1
R3-T8	pAB02^*a*^	-	10 (9)	GR12/29	AAR00517.1	AY228470.1
R3-T9	pABUH5-114	-	9 (9)	GR30	WP_000095317.1	NZ_AYOI01000002.1
R3-T10	p2ABSDF^*a*^	-	8 (8)	GR12	CAP02944.1	CU468232.1
R3-T11	p5ZQ3^*e*^	Aci4	8 (8)	GR4	PQJ03708.1	CM009651.1
R3-T12	p1ATCC19606	-	7 (6)	GR2	QFQ03441.1	CP045108.1
R3-T13	pABA-6973	-	7 (5)	-	AUT39979.1	CP026126.1
R3-T14	pAb242_9	-	5 (5)	GR4^*f*^	AUO31880.1	KY984045.1
R3-T15	pABAY15001_6E	-	5 (5)	-	QBN23345.1	MK386684.1
R3-T16	pTG31986	-	5 (5)	-	QCD20890.1	CP039342.1
R3-T17	pAba3207a	-	5 (5)	GR26	ANC38759.1	CP015365.1
R3-T18	p1ABAYE^*a*^	-	4 (4)	GR11	CAM84608.1	CU459137.1
R3-T19	pACICU1b^*a*^	Aci10	4 (4)	GR10	QCS03997.1	CP031381.2
R3-T20	pRCH51-3	-	4 (4)	-	AQT19035.1	KY216144.1
R3-T21	pABA-2f10	-	4 (4)	-	AUT40247.1	CP026129.1
R3-T22	pAb-MCR4.1	-	4 (4)	-	AYY91210.1	CP033872.1
R3-T23	p1ZQ8	-	4 (3)	-	PQL85636.1	CM009033.2
R3-T24	pK09-14	-	4 (4)	-	QER77251.1	CP043954.1
R3-T25	pD36-4	-	3 (3)	GR31	ALJ89824.1	CP012956.1
R3-T26	pAba810CPa	-	3 (3)	GR27	AXG87071.1	CP026340.1
R3-T27	pA52-2	-	3 (3)	-	QAB42494.1	CP034094.1
R3-T28	pDETAB2	-	3 (3)	GR34	QMS84089.1	CP047975.1
R3-T29	p2Res13-Abat^*g*^	-	3 (3)	-	QPF15404.1	CP062922.1
R3-T30	pPM193665_5	-	2 (2)	-	QJG86394.1	CP050420.1
R3-T31	pR2091	Aci5^*h*^	2 (0)	GR5	CUW37058.1	LN997847.1
R3-T32	pAb242_25^*i*^	Aci8^*i*^	2 (2)	GR8/23	AUO31913.1	KY984047.1
R3-T33	p3FDAARGOS_540	-	2 (1)	-	AYX85221.1	CP033751.1
R3-T34	pA1296_2	-	2 (2)	-	ATI40537.1	CP018334.1
R3-T35	pNaval81-26	-	2 (2)	GR28	WP_000185726.1	NZ_AFDB02000003
R3-T36	pAb242_25	-	2 (2)	GR22	AUO31910.1	KY984047.1
R3-T37	pAB1^*a*^	-	1	GR17	ABO13860.1	CP000522.1
R3-T38	p1ABSDF^*a*^	-	1	GR1	CAP02936.1	CU468231.1
R3-T39	p2ABSDF^*a*^	-	1	GR18	CAP02966.1	CU468232.1
R3-T40	p3ABSDF^*a*^	-	1	GR7	CAP02976.1	CU468233.1
R3-T41	p3ABSDF^*a*^	-	1	GR9	CAP02983.1	CU468233.1
R3-T42	p3ABSDF^*a*^	-	1	GR15	CAP02992.1	CU468233.1
R3-T43	pRCH52-1	Aci7^*j*^	1	GR3	ALC76579.1	KT346360.1
R3-T44	pFDAARGOS_540	-	1	-	AYX85290.1	CP033753.1
R3-T45	p29FS20-1	-	1	-	QLF12435.1	CP044520.1
R3-T46	pEH_gr13	-	1	-	QBR82727.1	CP038259.1
R3-T47	pE47_009	-	1	-	-^*k*^	CP042565.1
R3-T48	p1Res13-Abat^*g*^	-	1	-	QPF15412.1	CP062923.1
R3-T49	pABUH2a-5.6	-	1	-	WP_032021082.1	NZ_AYFZ01000080
R3-T50	pA1429b	-	1	-	QLB37617.1	CP046900.1
R3-T51	p4ZQ2	-	1	-	PQJ03811.1	CM009050.2
R3-T52	pDT0544C	-	1	-	QLI38221.1	CP053216.1
R3-T53	pDA33382-2-2	-	1	-	AXB17573.1	CP030107.1
R3-T54	pP7774	-	1	-	QCR91187.1	CP040262.1
R3-T55	pE47_008	-	1	-	QFH47722.1	CP042564.1
R3-T56	pVB2486_5	-	1	-	QJH05174.1	CP050408.1
R3-T57	pAba10324a	-	1	-	AXX46897.1	CP023023.1
R3-T58	pA52-4	-	1	-	QAB42527.1	CP034096.1
R3-T59	pOCU_Ac16a_3	-	1	-	-^*k*^	AP023080.1
R3-T60	p2ZQ2	-	1	-	PQJ03830.1	CM009647.1
R3-T61	pAC1633-4	-	1	-	QOJ62393.1	CP059302.1
R3-T62	pS30-1	-	1	-	ARH59503.1	KY617771.1
R3-T63	pD36-4	-	1	-	ALJ89842.1	CP012956.1
R3-T64	pDT01139C	-	1	-	QLI41769.1	CP053221.1
R3-T65	pKCRI-43-1	-	1	-	-^*k*^	LR026973.1
R3-T66	pE47_003	-	1	-	QFH47692.1	CP042559.1
R3-T67	pAC1633-2	-	1	-	QOJ62400.1	CP059303.1
R3-T68	pAb242_12	-	1	GR21	AUO31881.1	KY984046.1
R3-T69	p135040	-	0^*l*^	GR19	ACX70400.1	GQ861437.1

a Plasmids used in Bertini et al., 2010.

b Groups assigned by Bertini et al., 2010 (GR1-GR19), Lean (GR20), Cammeranesi et al., 2020 (GR21-GR23), Salgardo Camargo (GR24-GR33) and Liu et al., 2020 (GR34). Brackets indicate types falling within the GR guidelines at 74% *rep* gene identity.

c Bertini et al., 2010 included four Aci1 plasmids pAB2, pACICU1, p2ABAYE and pAB0057. pA1-1 included as the oldest known example.

d Plasmid pD72-1 replaces pABV01 used by Bertini et al., 2010, which is now known to be a hybrid of the majority of Aci1 and Aci2 sequences.

e Partial sequence only in Bertini et al., 2010 (p844; GU978998).

f Recorded as GR4 in Cammeranesi et al., 2020 and is 90.79% identical to the GR4 reference.

g Unnamed plasmids named here. Full strain names are p1Res13-Abat-PEA21-P4-01-A and p2Res13-Abat-PEA21-P4-01-A.

h Representative *rep* is in a partial sequence only (p537; GU978999) in Bertini et al., 2010.

i *rep* of pAb242_25 (encoding AUO31913.1) is identical to a partial sequence (p11921; GU979000) corresponding to Aci8 in GR8 in Bertini. et al., 2010. Also assigned Aci23 in Cammeranesi et al., 2020.

j pRCH52-1 *rep* is 95.16% identical to *rep*Aci7 (p11921; GU978996) from a partial sequence. No complete plasmids with identical *rep* genes were detected.

k Not available; sequence file not annotated.

l No complete plasmids correspond to a *rep* in a partial of p135040 (GQ861437) in Bertini. et al., 2010.

**FIG 3:**
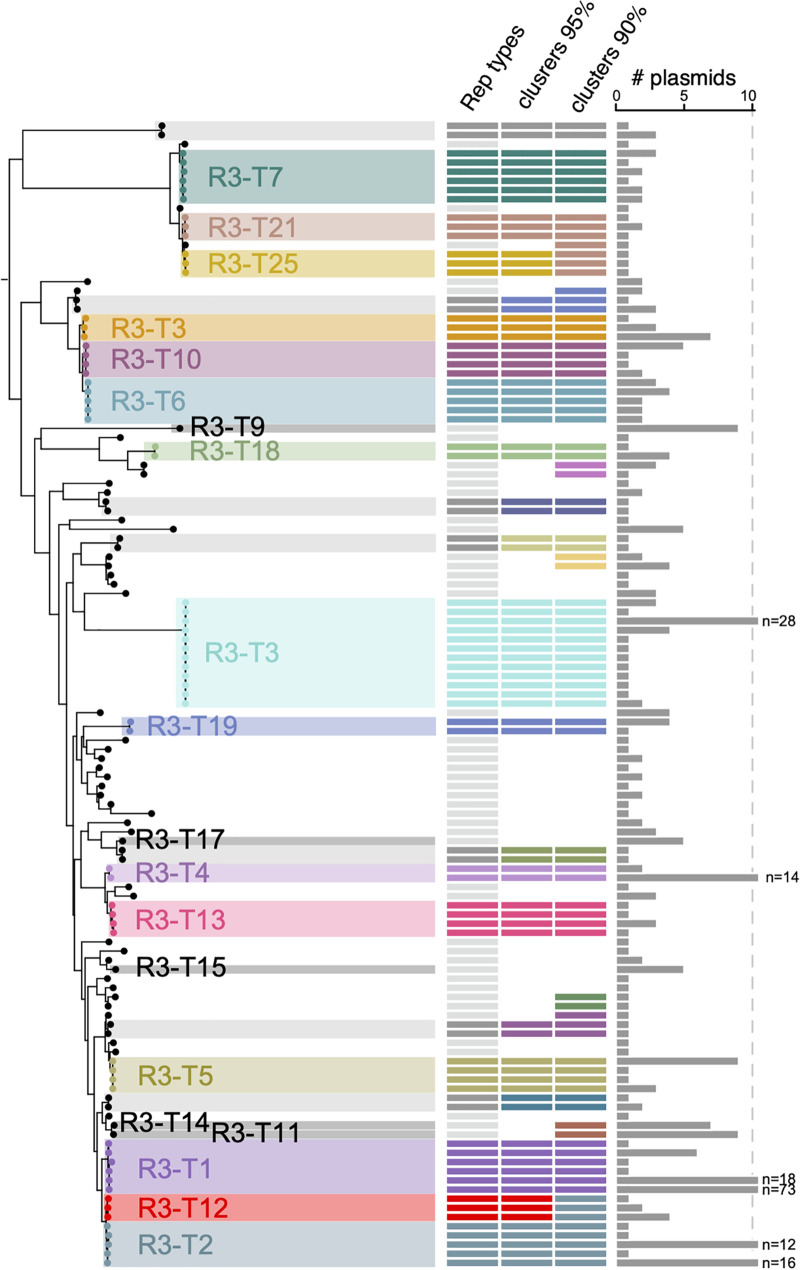
Maximum-likelihood phylogenetic relationship of replication initiation Rep sequences from the Rep_3 family. The tree comprises amino acid sequences encoded by *n* = 125 unique nucleotide sequence variants from the Rep_3 family generated in IQ-TREE. The shading show either sequence variants assigned to the same Rep3 family (unlabeled) or those detected in at least *n* = 5 plasmids (labeled). Columns are as follows: Rep types assigned based on a threshold of 95% nucleotide identity (those in light gray correspond to unique Rep types with a single nucleotide variant), clusters of Rep sequence variants grouped at 95% and 90% identity assigned with CD-HIT. The number of plasmids in which each Rep-encoding sequence variant was detected is shown on the right-hand side.

In some cases, recombination has occurred between related types generating hybrid *rep* genes that complicate classification. Our detailed examination of the *rep* genes of a number of plasmids included in the same R3 type but that have <99% identity overall to the type representative revealed that they consisted of 2 or more segments, the largest segment with very high identity to the representative for the assigned type and the remainder closely matching another type representative. One example is the *rep* genes of pAB02 and pABIR from the original GR12 group where the pABIR *rep* differs from the *rep* in the remaining members of the group with differences clustered at the 3′-end. A further example is the *rep*Aci1/*rep*Aci2 hybrid found in pABVA01 (Fig. 2B), which encodes the Aci2 exemplar in the original classification. The phylogeny of the Rep proteins shown in Fig. 3 illustrates how some types, for example R3-T21 and R3-T25 or R3-T2 and R3-T12, would amalgamate if the cutoff used were reduced to 90% protein sequence identity.

Plasmids encoding a Rep_3 family replication initiation protein are usually associated with iterons and use a theta replication mode. Indeed, iterons were identified in most of the Rep_3 encoding plasmids identified in the original classification (see Table 6). However, the presence of iterons was not systematically examined here.

### GR2 includes the R3-T1, R3-T2 and R3-T12 groups.

An examination of the relationships between members of the R3-T1, R3-T2 and R3-T12 groups, which would all be included in the original GR2 group (2) provides insight into the reasons for increasing the cutoff to 95% identity. Most R3-T1 plasmids (encoding the Aci1 Rep protein) are found in multiply, extensively and pan resistant isolates belonging to global clones GC1 and GC2 which dominate the total genome sequences available currently. Indeed, the plasmid pA1-1 (GenBank accession number CP010782; [37]), which is found in the oldest GC1 isolate (1982) for which sequence data is available, is identical to pAB0057 (2004), indicating a long association of the plasmid with the clone. Many plasmids identical or nearly identical to pAB0057/pA1-1 were found among available complete sequences in a previous analysis (16). About half of the members of the R3-T1 group reported here are identical or nearly identical to pA1-1 with differences in recorded length generally due to failure to trim the overlap from linear contigs. The pAB0057/pA1-1 plasmid consists of a backbone and 2 *dif* modules (Fig. 2A), though this was not initially recognized. Indeed, further R3-T1 plasmids carrying the same *rep*Aci1 gene have the same backbone but a different *dif* module in the first position or have a different set of *dif* modules, e.g., p2ABAYE and pD36-3 (Fig. 2A). This lineage was also among the most abundant identified by others (10).

The *rep* gene encoding RepAci2 found in the R3-T2 type, also originally classified as GR2, is only approximately 80% identical to RepAci1 and was re-assigned to GR20 (5). However, here a third clearly distinct *rep* type (92.7% identity to *rep*Aci2) that would fall into the original GR2 group was found in p1ATCC19606 (38). This type was designated R3-T12. The close relationship between these 3 types can be seen in the phylogeny of the R3 Rep proteins (Fig. 3).

### Acinetobacter Plasmid Typing database.

To enable others to detect the presence of the Acinetobacter plasmid *rep* types defined here, we have made available databases comprising reference nucleotide sequences in GitHub (https://github.com/MehradHamidian/AcinetobacterPlasmidTyping). The databases comprise simple multi-fasta files that include a representative sequence for each *rep* type with information on the type plasmid (name and GenBank accession number) provided in the header. These files can be used to screen genome assemblies using BLASTN (run locally or using web-based services to screen read sets), or SRST2 (39) (https://github.com/katholt/srst2) using a threshold of 95% nucleotide identity to match the threshold used to define *rep* types. The databases can also be used to screen sequencing reads for *rep* types, facilitating the detection of plasmid-derived contigs in short read assemblies for which no tool is currently available for Acinetobacter.

### Use of Acinetobacter plasmid typing database for read-based analysis.

To demonstrate the use of the databases for detection of plasmids in genome data, short read data sets used previously in an analysis of a collection of 41 GC1 isolates (40) were examined (see Materials and Methods). Analysis of the read data for the 36 isolates for which complete genomes were not available revealed that all but one of the isolates in the collection included a detectable plasmid *rep* gene (Table 8). Plasmids present in available complete genomes of 5 isolates (strain name shown in bold in Table 8) are also listed.

**TABLE 8:** Distribution of plasmid Rep types in GC1 isolates studied in Holt et al., 2016

Genomes	Date	Country	ST	GenBank no.^*b*^	R3-T	RP-T	R1-T
A1^*a*^	1982	UK	1	CP010781-2	T1^*c*^	-	-
A388^*a*^	2002	Greece	1	CP024418-9	T1, T19^*d*^	-	-
AB0057^*a*^	2004	USA	1	CP001182-3	T1^*e*^	-	-
A85^*a*^	2003	Australia	1	CP021782-7	T1^*f*^	T1	T1, T2
A297	1984	Netherlands	1	FBWR	T1	-	-
J1	1995	Australia	1	FBWQ	T1	T1	-
J5	1997	Australia	1	FBWP	T1	T1	-
WM98	1998	Australia	1	UCPN	T1	-	-
J7	1998	Australia	1	FBWT	T1	-	-
J10	1999	Australia	1	FBWS	T1, T2	-	-
D3208	1997	Australia	1	FBWZ	T1	-	-
D2	2006	Australia	1	FBWY	T1, T15	-	-
D62	2010	Australia	1	FBWW	T1	-	-
D30	2008	Australia	1	FBXG	T1	-	-
A83	2002	Australia	1	FBWU	T1	-	-
A92	2005	Australia	1	FBWV	T1	-	-
6772166	2002	Australia	1	FBWX	T1	-	T2
RBH3	2002	Australia	1	FBXD	T1	-	T2
D13	2009	Australia	1	FBXI	T14	-	-
D15	2009	Australia	1	FBXJ	T14	-	-
G7	2003	Australia	1	FBXF	T1	T1	-
D81	2010	Australia	1	FBXC	T19	T1	-
D78	2010	Australia	1	FBXH	T19	T1	-
Naval-83	2006	USA	20	AMFK	-	T1	-
AB058	2003	USA	20	ADHA	T1, T8	-	-
NIPH 527	1984	Netherlands	1	APQW	T1	-	-
NIPH 290	1994	Netherlands	1	APRD	T9	-	-
AB056	2004	USA	1	ADGZ	T1	-	T2
AB059	2004	USA	1	ADHB	T1	T1	-
908-13	2007	USA	1	AMHW	-	-	-
909-02-7	2007	USA	1	AMHZ	T1	-	-
Canada-BC1	2007	Canada	1	AMSZ	-	-	-
Canada-BC5	2007	Canada	1	AFDN	T1	-	-
IS-58	2008	nr	1	AMGH	-	T1	-
ANC 4097	2011	nr	1	APRF	T1, T12, T57	T1	T4
D36^*a*^	2008	Australia	81	CP012952-6	T1, T25, T63^*g*^	-	-
6013113	2007	UK	81	ACYR	T25, T63	-	-
6013150	2007	UK	81	ACYQ	T1, T4, T25, T63	-	-
OIFC074	2003	USA	19	AMDE	T1	-	-
Naval-21	2006	USA	19	AMSY	-	T1	-
TG19582	nr	nr	1	AMIV	T63	-	-

a Complete genomes.

b All draft genome accession numbers include “00000000”.

c Encoded by pA1-1 (GenBank accession number CP010782).

d Both R3-T1 and R3-T19 Reps are encoded by pA388 (GenBank accession number CP024419).

e Encoded by pAB0057 (GenBank accession number CP001183).

f A85 carries 5 plasmids, pA85-1 (R1-T1), pA85-1a (R1-T2), pA85-1b (does not encode a Rep), pA85-2 (R3-T1) and pA85-3 (RP-T1).

g D36 carries 4 plasmids. pD36-1 and pD36-2 (pRAY*) (GenBank accession numbers CP012953.1 and CP012954.1, respectively) do not encode a Rep. pD36-3 (GenBank accession number CP012955.1) encodes a R3-T1 Rep and pD36-4 (GenBank accession number CP012956.1) encodes two Reps (R3-T25 and R3-T63).

An R3-T1 plasmid was detected in most isolates, including all five with a completed genome. Inspection of the relevant contigs revealed that in all isolates belonging to GC1 lineage 1, this plasmid was identical or nearly identical to pAB0057/pA1-1. However, in the ST81 group in lineage 2 (isolates D36, 6013113 and 6013150) the R3-T1 plasmid matched pD36-3 which includes different *dif* modules. Plasmid types R3-T25 and R3-T63 were also found in ST81 isolates 6013113 and 6013150 consistent with their presence in the complete genome of D36, as reported previously (17).

A R3-T19 (Aci10) type plasmid was detected in isolates D78 and D81 together with an RP-T1 (Aci6) plasmid. The RP-T1 plasmids were previously reported to be present and identical to pAb-G7-2 (40), and this was verified using a complete genome assembly of D78 (C. J. Harmer and R. M. Hall, unpublished). The R3-T19 plasmid in D78 was found to include the *oxa58* carbapenem resistance gene. Similarly, the presence of an R3-T14 type in the related isolates D13 and D15 was confirmed using a complete genome assembly for D13 (C. J. Harmer and R.M. Hall, unpublished). However, the complete D13 genome assembly included a further Rep_3 plasmid that was of a type not present in the current database, indicating that updates to the plasmid typing scheme will be needed in the future.

## DISCUSSION

The analyses described here enabled development of a simple classification scheme which covers the plasmids found in A. baumannii that include a *rep* gene encoding an identifiable replication initiation or Rep protein. The cutoff 95% nucleotide identity was chosen because it is readily able to accommodate minor sequence difference arising from evolution or from sequencing errors and also facilitates grouping of hybrid genes with the one type from which the majority of its sequence has been derived. The effect of raising the cutoff is clear in Fig. 3 where groups of 3 coherent types can be found within the former GR2 (R3-T1, R3-T2 and R3-T12) and within the former GR12 (R3-T6, R3-T8 and R3-T10).

The simple strategy applied here provides a framework for the research community to classify A. baumannii plasmids as the simple rules will allow new plasmid types to be added to the scheme as they are discovered. Ultimately, this may include some of the known large conjugative plasmid families which likely encode a Rep protein of a type that has not yet been identified and verified experimentally and hence has not been assigned a Pfam. Once a potential *rep* gene is identified, these can be simply added into the scheme. This scheme should also be applicable to plasmids found in other Acinetobacter species as plasmid sharing is known to occur (14).

Because there is little variation between the *rep* genes in members of individual types in the R1 and RP groups and significant variation between the *rep* gene associated with each type, identification of additional types should be straightforward. Indeed, complete sequence of plasmids belonging to each R1 type have so far been identical. In the case of the RP-T1 and RP-T2 groups, members share very closely related backbones. However, in the case of the more diverse R3 group, the effect of recombination between moderately closely related plasmids may lead to complications in the future. One prime example is the hybrid *rep* genes that arise from recombination of 2 or more types potentially leading to formation of *rep* types that do not fit neatly into an existing category. Indeed, some examples of hybrids were identified here among plasmids with backbones of the iteron-*rep*-orfX configuration that include *dif* modules. However, a more detailed analysis of the more diverged members of the R3 types that include plasmids with *rep* genes that are less than 99% identical to the type representative will be needed to further explore these relationships. For this group, an improvement in the accuracy of annotation of *rep* genes is clearly needed. However, we note that in the RefSeq version of these plasmid sequences, the validated *rep* gene is annotated as *repM*, following Dorsey et al. (35) nomenclature, and orfX is annotated as a helix-turn-helix domain protein. The Aci classifications (*rep*Aci) that are applicable to some of the dominant types have been widely used and are likely to remain useful into the future.

The sequence files containing representative sequences of each type have been made available in a public GitHub repository and will simplify classification of competed plasmid genomes. These files will underpin the detection of plasmids in short read DNA sequence data, which is rarely undertaken currently due to the difficulties involved. This should lead to a more comprehensive approach to the surveillance of the role of plasmid-encoded functions in antibiotic resistance, virulence, and environmental survival.

## MATERIALS AND METHODS

### Plasmid sequence data collection.

Plasmids from A. baumannii whole genome sequence projects were downloaded from GenBank (https://www.ncbi.nlm.nih.gov/genbank/) in February 2021 and duplicate sequences from the same isolate were removed. Plasmids with sequences of poor quality were eliminated for various reasons such as sequencing errors, assembly issues, circularisation/trimming issues or the *rep* gene was incomplete. Reasons for removal are documented in Table S1. After curation, there were 539 plasmid nucleotide sequences available from published genome projects.

To capture complete plasmids sequenced using traditional methods (not part of a genome project), the RefSeq (https://www.ncbi.nlm.nih.gov/refseq/) database was also searched using the search terms “Acinetobacter baumannii AND complete AND plasmid and srcdb_refseq[PROP]”. All plasmids with accession numbers starting with CP, CM, CU etc. indicating they were from complete genomes, were removed and the remainder curated as described above. This identified an additional 81 plasmids (including the 15 complete plasmids and 1 partial sequence described in [2] now completed), resulting in a final data set comprising 621 complete plasmids. This data set was used to develop the typing scheme in this study.

Initially, plasmid entries with annotations were inspected manually, by searching for words such as replication or rep, to find any annotated *rep* genes. In the case of plasmids encoding a Rep_3 family Rep, the genes incorrectly annotated as *repA* were identified (see Results for details) and removed from the gene set. The remaining entries with no annotations or with no annotated *rep*/REP were initially further screened using low stringency BLASTn searches with each of the DNA sequences of *rep* genes that had been previously described in Bertini et al., (2). To find *rep* genes in the remainder of entries, they further examined using tBLASTn using the amino acid sequences of Rep proteins described in (2). In addition searches with the complete sequences or the backbone sequences of the plasmids previously found to be devoid of a *rep* gene including pRAY* (GenBank Accession number CP012954.1), pA85-1b (GenBank Accession number CP021785.1), pD36-1 (GenBank Accession number CP012953.1), pA297-3 (GenBank accession number), pNDM-BJ01 (GenBank accession number JQ001791.1), and pALWED1.1 (GenBank accession number CP082144.1) were used to identify related plasmids.

After identification of the plasmids encoding a Rep protein, the DNA sequences of *rep* genes identified using annotations, BLASTn and tBLASTn were extracted and used to create a local database used for further analysis of the *rep* genes. Amino acid sequence data for the Rep of each entry were extracted and used to populate a second local database used for further analysis.

### Clustering and phylogenetic analysis of the *rep*/Rep sequence data.

Clusters comprising *rep* sequences at >80%, >85%, >90% and >95% nucleotide similarity were derived using CD-HIT Suite (https://github.com/weizhongli/cdhit) (41). To study the relationships of the Rep protein sequences, the nucleotide sequences for the extracted *rep* sequences were translated to amino acid sequences with EMBOSS Transeq and aligned with MUSCLE v3.8.31 (42). The aligned sequence file was used to infer a maximum-likelihood phylogeny with IQ-TREE version 1.6.10 (43, 44) with the VT+F+G4 model (selected from the -m test model selection flag) with 100 bootstrap replicates. The final tree was visualized in FigTree v1.4.4 (http://tree.bio.ed.ac.uk/), and tree annotation generated with the plotTree code (github.com/katholt/plotTree) in R v1.1.456.
