# Supplementary material for: Detection and Typing of Plasmids in Acinetobacter baumannii Using rep Genes Encoding Replication Initiation Proteins
Source: Microbiol Spectr. 2022 Dec 6;11(1):e02478-22. doi: 10.1128/spectrum.02478-22 (PMC9927589; doi:10.1128/spectrum.02478-22)
Supplement: Supplemental file 1 — Supplemental material. Download spectrum.02478-22-s0001.pdf, PDF file, 0.4 MB [file spectrum.02478-22-s0001.pdf]

Table S1 Plasmids excluded from study

Accession number	Reason for exclusion
CP050525.1	Not plasmid sequence – RNA
CP040042.1	Not plasmid sequence – Chromosome
CP040041.1	Not plasmid sequence – Chromosome
CP040048.1	Not plasmid sequence – Chromosome
CP020583.1	Contaminated sample
CP016296	Duplicated accession number
CP016297	Duplicated accession number
CP016299	Duplicated accession number
CP016301	Duplicated accession number
CP016302	Duplicated accession number
CP017655	Duplicated accession number
CP047974	Duplicated accession number
CP047975	Duplicated accession number
CP047976	Duplicated accession number
KP890934.1	Assembly issue - Frameshift
CP035931.1	Assembly issue - Frameshift
CP033517.1	Assembly issue - Frameshift
NZ_AFDA02000007.1	Assembly issue - Frameshift
CP030084.1	Assembly issue - Frameshift
CP032741.1	Assembly issue - Frameshift
HG977528.1	Assembly issue - Frameshift
CP033519.1	Assembly issue - Frameshift
NZ_AFCZ02000004.1	Assembly issue - Frameshift
CP035674.1	Assembly issue - Frameshift
CP035675.1	Assembly issue - Frameshift
CP040046.1	Assembly issue - Frameshift
CP033518.1	Assembly issue - Frameshift
CP040081.1	Assembly issue - Frameshift
CP061542.1	Assembly issue - Frameshift
CP020575.1	Assembly issue - Frameshift
CP040082.1	Assembly issue - Frameshift
CP018862.2	Assembly issue - Gap
CP065393.1	Assembly issue - Gap
CP032216.1	Incomplete plasmid sequence
CP032219.1	Incomplete plasmid sequence
AP022837.1	Reassembled/ resubmitted plasmid
AP022838.1	Reassembled/ resubmitted plasmid
CP000865.1	Reassembled/ resubmitted plasmid
CP019218.1	Reassembled/ resubmitted plasmid
CP035052.1	Reassembled/ resubmitted plasmid
CP039024.1	Reassembled/ resubmitted plasmid
CP039026.1	Reassembled/ resubmitted plasmid
CP039027.1	Reassembled/ resubmitted plasmid
CP039029.1	Reassembled/ resubmitted plasmid
CP039030.1	Reassembled/ resubmitted plasmid
CP046655.1	Reassembled/ resubmitted plasmid
CP049364.1	Reassembled/ resubmitted plasmid
CP049365.1	Reassembled/ resubmitted plasmid
CP050398.1	Reassembled/ resubmitted plasmid
CP053099.1	Reassembled/ resubmitted plasmid
CP053100.1	Reassembled/ resubmitted plasmid
CP059042.1	Reassembled/ resubmitted plasmid

JQ904627.1	Reassembled/ resubmitted plasmid
KJ493819.2	Reassembled/ resubmitted plasmid
MK360916.1	Reassembled/ resubmitted plasmid
CP000864.1	Reassembled/ resubmitted plasmid
CP015485.1	Assembly issue - incomplete
CP050399.1	Plasmid segment
CP050396.1	Plasmid segment
CP050397.1	Plasmid segment
MK134375.1	Assembly issue – shortened *rep*
CP042995.1	Wrong species
CP042996.1	Wrong species
CP045561.1	Wrong species
CM009040.2	Assembly issue – Chromosomal contig
CM009044.2	Assembly issue – Chromosomal contig
CM009649.1	Assembly issue – Chromosomal contig
CP003907.1	Circularised genomic island
CP003908.1	Circularised genomic island
CM008887.1	Circularised genomic island
CP007579.1	Circularised genomic island
CP038646.1	Circularised genomic island
HG977524.1	Assembly issue – shortened *rep*
CP027184.1	Assembly issue – shortened *rep*
CP017643.1	Assembly issue – shortened *rep*
CM008887.2	Assembly issue – shortened *rep*

Table S2 Properties of RepPriCT_1 plasmids

Acession number	Biosample	strain_name	Plasmid name	Length(bp)	Rep_family	Type	Bertini scheme	Locus/Protein_id
AEOY01000095.1	SAMN02471598	3990	p1ABST2	63320	Rep	RP-T1	Aci6	-
AFDB02000004.1	SAMN00114928	Naval-81	pNaval81-67	67012	Rep	RP-T1	Aci6	EJP56764.1
AFDL01000006.1	SAMN02436631	OIFC143	pOIFC143-70	69518	Rep	RP-T1	Aci6	EJG16496.1
ALII01000020.1	SAMN02436485	IS-123	pIS123-67	67025	Rep	RP-T1	Aci6	EJO37624.1
AP023078.1	SAMD00059694	OCU_Ac16a	pOCU_Ac16a_1	73028	Rep	RP-T1	Aci6	-
AYOH01000010.2	SAMN02597375	UH9907	pABUH1-74	74089	Rep	RP-T1	Aci6	ETR49020.1
CM009084.2	SAMN08043814	ZQ1	p2ZQ9	62339	Rep	RP-T1	Aci6	PLK14912.1
CM009650.1	SAMN08093362	ZQ3	p4ZQ3	68115	Rep	RP-T1	Aci6	PQJ03752.1
CP001922.1	SAMN02603494	1656-2	ABKp1	74451	Rep	RP-T1	Aci6	ADX05416.1
CP002524.1	SAMN02603891	TCDC-AB0715	p2ABTCDC0715	70894	Rep	RP-T1	Aci6	ADX94329.1
CP007580.1	SAMN02471421	AC30	pAC30c	71433	Rep	RP-T1	Aci6	AHX67284.1
CP008707.1	SAMN02894434	AB5075-UW	p1AB5075	83610	Rep	RP-T1	Aci6	AKA33572.1
CP008851.1	SAMN02714232	AC29	pAC29b	74749	Rep	RP-T1	Aci6	AKB09352.1
CP012008.1	SAMN03817045	Ab04-mff	pAB04-2	87569	Rep	RP-T1	Aci6	AKQ32652.1
CP014216.1	SAMN04448797	YU-R612	p2YU-R612	74241	Rep	RP-T1	Aci6	AMC17795.1
CP014292.1	SAMN04457967	AB34299	p2AB34299	84967	Rep	RP-T1	Aci6	AQU58831.1
CP015366.1	SAMN04485290	3207	pAba3207b	80546	Rep	RP-T1	Aci6	ANC38839.1
CP016297.1	SAMN04096368	CMC-CR-MDR-Ab4	pCMCVTAb2-Ab4	74090	Rep	RP-T1	Aci6	APQ87400.1
CP016302.1	SAMN04096370	CMC-CR-MDR-Ab66	pCMCVTAb2-Ab66	73188	Rep	RP-T1	Aci6	APQ95054.1
CP017645.1	SAMN05601667	KAB02	pKAB02	81227	Rep	RP-T1	Aci6	AOX75640.1
CP017647.1	SAMN05601668	KAB03	pKAB03	73891	Rep	RP-T1	Aci6	AOX79427.1
CP017649.1	SAMN05601669	KAB04	pKAB04	121485	Rep	RP-T1	Aci6	AOX83318.1
CP017651.1	SAMN05601670	KAB05	pKAB05	70884	Rep	RP-T1	Aci6	AOX87285.1
CP017653.1	SAMN05601671	KAB06	pKAB06	70884	Rep	RP-T1	Aci6	AOX91169.1
CP017655.1	SAMN05601672	KAB07	pKAB07	72073	Rep	RP-T1	Aci6	AOX94964.1
CP017657.1	SAMN05601673	KAB08	pKAB08	101406	Rep	RP-T1	Aci6	AOX98817.1
CP019115.1	SAMN06211572	MDR-CQ	pMDR-CQ	75390	Rep	RP-T1	Aci6	QDR95729.1
CP020573.1	SAMN06650264	15A5	p15A5_1	74241	Rep	RP-T1	Aci6	ARF91039.1
CP020577.1	SAMN06650263	SSA12	pSSA12_1	73264	Rep	RP-T1	Aci6	ARF94730.1
CP020580.1	SAMN06650261	SSMA17	pSSMA17_1	90973	Rep	RP-T1	Aci6	ARG03955.1
CP020582.1	SAMN06650260	JBA13	pJBA13_1	90972	Rep	RP-T1	Aci6	ARG07627.1
CP020589.1	SAMN06650243	15A34	p15A34_1	72076	Rep	RP-T1	Aci6	ARG18954.1
CP020593.1	SAMN06650241	USA2	pUSA2_1	74241	Rep	RP-T1	Aci6	ARG29677.1
CP020594.1	SAMN06650240	USA15	pUSA15_1	98301	Rep	RP-T1	Aci6	ARG29717.1
CP021322.1	SAMN07135563	XH731	pXH731	64557	Rep	RP-T1	Aci6	AWW87056.1
CP021787.1	SAMN07125723	A85	pA85-3	86334	Rep	RP-T1	Aci6	ASF79226.1
CP023028.1	SAMN07520233	10042	pAba10042b	110728	Rep	RP-T1	Aci6	AXX50773.1
CP023030.1	SAMN07520232	9102	pAba9102a	95206	Rep	RP-T1	Aci6	AXX54541.1
CP023033.1	SAMN07284119	7847	pAba7847b	80546	Rep	RP-T1	Aci6	AXW92641.1
CP024578.1	SAMN07945345	AbPK1	pAbPK1b	79335	Rep	RP-T1	Aci6	ATR89566.1
CP025267.1	SAMN08054861	SMC_Paed_Ab_BL01	pSMC_AB_BL01_1	74241	Rep	RP-T1	Aci6	AUG14723.1
CP026706.1	SAMN04014897	AR_0056	tig00000059_pilon	72105	Rep	RP-T1	Aci6	AVE44386.1
CP026712.1	SAMN04014904	AR_0063	unitig_2_pilon	72990	Rep	RP-T1	Aci6	AVE56836.1
CP026946.1	SAMN07977762	S1	pAbS1_03	72204	Rep	RP-T1	Aci6	AVG28625.1
CP027121.1	SAMN04014897	AR_0056	p2AR_0056	72105	Rep	RP-T1	Aci6	AVN03791.1
CP027243.1	SAMN08364584	WCHAB005078	p1_005078	70796	Rep	RP-T1	Aci6	AVN12622.1
CP029571.1	SAMN09241862	DA33098	pDA33098-71	71234	Rep	RP-T1	Aci6	AWO18521.1
CP030109.1	SAMN09460321	DA33382	pDA33382-85	84678	Rep	RP-T1	Aci6	AXB17624.1
CP031382.1	SAMN09302593	ACICU	pACICU2	70101	Rep	RP-T1	Aci6	QCS04021.1
CP033245.1	SAMN07520235	7835	pAba7835b	80584	Rep	RP-T1	Aci6	QFY70971.1
CP035047.1	SAMN05238672	ABUH793	p74.1Kbp	74091	Rep	RP-T1	Aci6	QAS99722.1
CP036286.1	SAMN10261590	TG60155	p60155_3	71234	Rep	RP-T1	Aci6	QBH55839.1
CP038264.1	SAMN10386510	LEV1449/17Ec	pEC_gr6	76956	Rep	RP-T1	Aci6	QBR79239.1
CP039994.1	SAMN10261544	TG22182	pTG22182_1	71233	Rep	RP-T1	Aci6	QCO84474.1
CP040043.1	SAMN11554497	VB958	p3VB958	82500	Rep	RP-T1	Aci6	QCP18569.1
CP042842.1	SAMN12399660	ATCC BAA-1790	pATCCBAA-1790	67023	Rep	RP-T1	Aci6	QEE59151.1
CP050392.1	SAMN14410107	VB11737	pVB11737_1	70712	Rep	RP-T1	Aci6	QJH08823.1
CP050404.1	SAMN14414761	VB2486	pVB2486_1	99090	Rep	RP-T1	Aci6	QJH05026.1
CP050413.1	SAMN14420253	PM192696	pPM192696_1	70098	Rep	RP-T1	Aci6	QJG90047.1
CP050909.1	SAMN14308866	DT-Ab022	p2DT-Ab022	71194	Rep	RP-T1	Aci6	QIX36610.1
CP050912.1	SAMN14308864	DT-Ab020	p2DT-Ab020	71194	Rep	RP-T1	Aci6	QIX40477.1
CP050915.1	SAMN14308853	DT-Ab007	pDT-Ab007	92021	Rep	RP-T1	Aci6	QIX44206.1
CP050917.1	SAMN14308849	DT-Ab003	p2DT-Ab003	71194	Rep	RP-T1	Aci6	QIX48008.1
CP061516.1	SAMN12391854	CFSAN093710	pCFSAN093710_2	83626	Rep	RP-T1	Aci6	QNV20001.1
CP062921.1	SAMN16304032	Res13-Abat-PEA21-P4-01-A	p3Res13-Abat	72831	Rep	RP-T1	Aci6	QPF15346.1
CP066230.1	SAMN17073618	G20AB011	pG20AB011-1	73339	Rep	RP-T1	Aci6	QQD98159.1
CP066233.1	SAMN17073617	G20AB010	pG20AB010-1	73360	Rep	RP-T1	Aci6	QQD94357.1
CP066236.1	SAMN17073616	G20AB009	pG20AB009-1	73361	Rep	RP-T1	Aci6	QQE05740.1
CP066238.1	SAMN17073615	G20AB007	pG20AB007-1	73361	Rep	RP-T1	Aci6	QQE01989.1
CP069841.1	SAMN16357502	FDAARGOS_1360	p2FDAARGOS_1360	76933	Rep	RP-T1	Aci6	QRR53484.1
CP072124.1	SAMN18396008	KSK1	p2KSK1	87529	Rep	RP-T1	Aci6	QTH58400.1
CP072272.1	SAMN18452234	KSK6	p2KSK6	68224	Rep	RP-T1	Aci6	QTK45694.1
CP072277.1	SAMN18452334	KSK7	p2KSK7	68225	Rep	RP-T1	Aci6	QTK62065.1
CP072282.1	SAMN18452342	KSK10	p2KSK10	87529	Rep	RP-T1	Aci6	QTK53937.1
CP072287.1	SAMN18452659	KSK11	p2KSK11	87529	Rep	RP-T1	Aci6	QTK58013.1
CP072292.1	SAMN18452698	KSK18	p2KSK18	68225	Rep	RP-T1	Aci6	QTK66135.1
CP072297.1	SAMN18452699	KSK19	p2KSK19	87529	Rep	RP-T1	Aci6	QTK70246.1
CP072302.1	SAMN18452712	KSK20	p2KSK20	68309	Rep	RP-T1	Aci6	QTK74363.1
CP072400.1	SAMN18451305	KSK2	p2KSK2	87529	Rep	RP-T1	Aci6	QTL10322.1
HG977523.1	SAMEA3158456	CS01	pCS01A	63720	Rep	RP-T1	Aci6	-
HG977527.1	SAMEA3158506	CR17	pCR17A	63795	Rep	RP-T1	Aci6	-
KF669606.1	SAMN14225999	G7	pAb-G7-2	70100	Rep	RP-T1	Aci6	AGY56182.1
KF889012.1	SAMN02603667	TYTH-1	pAB_CC	65890	Rep	RP-T1	Aci6	AII26481.1
KM051846.1	SAMN14226465	D72	pD72-2	70102	Rep	RP-T1	Aci6	AIH07963.1
KM977710.1	SAMN14226502	D46	pD46-3	74916	Rep	RP-T1	Aci6	AIZ49259.1
KR535992.1	SAMN14226627	A105	pA105-1	70098	Rep	RP-T1	Aci6	ALN43326.1
KU549175.1	SAMN14227264	C13	pC13-2	103871	Rep	RP-T1	Aci6	AMQ95288.1
KX230794.1	SAMN14227145	MAL	pMAL-2	67025	Rep	RP-T1	Aci6	ANR95813.1
KY022424.1	SAMN14227393	Ab8098	pAb8098	82667	Rep	RP-T1	Aci6	-
LT984690.1	SAMEA104446236	K50	p2K50	79598	Rep	RP-T1	Aci6	SPC58401.1
MG954377.1	SAMN14228297	SGH9601	pS21-2	123432	Rep	RP-T1	-	AWO68424.1
MG954379.1	SAMN14228295	SGH0905	pS32-2	70833	Rep	RP-T1	Aci6	AWO68573.1
MK243454.1	SAMN14227902	09A16CRGN0014	pCRA914-67	66886	Rep	RP-T1	Aci6	QAR16211.1
MK386681.1	SAMN14228691	ABAY09008	pABAY09008_1B	74241	Rep	RP-T1	Aci6	QBN23166.1
MK531538.1	-	MC23	pMC23.1	67441	Rep	RP-T1	-	QCO89676.1
CP003501.1	SAMN02603104	MDR-TJ	pABTJ1	77528	Rep	RP-T2	-	-
CP003887.1	SAMN02604244	BJAB07104	p1BJAB07104	70170	Rep	RP-T2	-	-
CP003888.1	SAMN02604246	BJAB0868	p2BJAB0868	70167	Rep	RP-T2	-	-
CP018144.1	SAMN06046790	HRAB-85	pHRAB-85	77513	Rep	RP-T2	-	APF45706.1
CP018422.1	SAMN06109232	XDR-BJ83	pBJ83	69069	Rep	RP-T2	-	APM50974.1
KM922672.1	SAMN03103694	A221	pAZJ221	77530	Rep	RP-T2	-	-
MK386682.1	SAMN14228690	ABAY10001	pABAY10001_1C	54627	Rep	RP-T2	-	QBN23313.1
CP013925.1	SAMN03941550	KBN10P02143	pKBN10P02143	52517	Rep	RP-T3	-	ALY01450.1
CM009039.2	SAMN08093365	ZQ6	p2ZQ6	6772	Rep	RP-T4	-	PQL72098.1
CP042563.1	SAMN12289292	E47	pE47_007	4715	Rep	RP-T5	-	QFH47718.1
CP059390.1	SAMN15541804	36-1512	p4.36-1512	4721	Rep	RP-T5	-	QLY88352.1

Table S3 Rep_3 plasmids

Acession number	Biosample	strain_name	Plasmid name	Length(bp)	Rep_family	Type	Bertini scheme	Locus/Protein_id
CP000523.1	SAMN02604331	ATCC 17978	pAB2	11302	Rep_3	R3-T1	A1S_3472	ABO13861.1
CU459138.1	SAMEA3138279	AYE	p2ABAYE	9661	Rep_3	R3-T1	p2ABAYE0002	CAM84615.1
CP000523.1	SAMN02604331	ATCC 17978	pAB2	11302	Rep_3	R3-T1	Aci1	ABO13861.1
CP001183.2	SAMN02603051	AB0057	pAB0057	8731	Rep_3	R3-T1	Aci1	ACJ43223.1
CP001183.2	SAMN02603051	AB0057	pAB0057	8731	Rep_3	R3-T1	Aci1	ACJ43223.1
CP024419.1	SAMN07736509	A388	pA388	33036	Rep_3	R3-T1	Aci1	ATP89005.1
CP031381.2	SAMN09302593	ACICU	pACICU1b	24268	Rep_3	R3-T1	Aci1	QCS03991.1
CU459138.1	SAMEA3138279	AYE	p2ABAYE	9661	Rep_3	R3-T1	Aci1	CAM84615.1
MN266872.1	-	N/A	pAC1-BRL	16673	Rep_3	R3-T1	Aci1	QHW11277.1
AB823544.1	SAMN14229301	NCGM 253	pAB-NCGM253	8970	Rep_3	R3-T1	Aci1	-
AEOY01000096.1	SAMN02471598	3990	p2ABST2	21846	Rep_3	R3-T1	Aci1	-
AEOZ01000236.1	SAMN02471606	3909	p1ABST78	26411	Rep_3	R3-T1	Aci1	-
AEPA01000396.1	SAMN02471587	4190	p2ABST25	8970	Rep_3	R3-T1	Aci1	-
AFDN01000003.1	SAMN02436468	Canada BC-5	pCanadaBC5-8.7	8731	Rep_3	R3-T1	Aci1	EJO35917.1
AYEX01000118.1	SAMN06650245	CBA7	pABUH6a-8.8	8763	Rep_3	R3-T1	Aci1	ETR37079.1
CM003314.1	SAMN02906929	MRSN 7339	pMRSN7339-8.7	8731	Rep_3	R3-T1	Aci1	KLT75075.1
CM003317.1	SAMN02906928	MRSN 58	pMRSN58-8.7	8731	Rep_3	R3-T1	Aci1	KLT95674.1
CM003741.1	SAMN04407353	MEX11594	p2MEX11594	5557	Rep_3	R3-T1	Aci1	-
CM003909.1	SAMN03450127	AB210M	pAB0057	8781	Rep_3	R3-T1	Aci1	KZC87498.1
CM009038.2	SAMN08093365	ZQ6	p1ZQ6	8905	Rep_3	R3-T1	Aci1	PQL72050.1
CM009043.2	SAMN08093364	ZQ5	p2ZQ5	8731	Rep_3	R3-T1	Aci1	PQL83867.1
CM009924.1	SAMN07602915	CCUG 70743	pAba70743_1	10880	Rep_3	R3-T1	Aci1	PXF35729.1
CP002523.1	SAMN02603891	TCDC-AB0715	p1ABTCDC0715	8731	Rep_3	R3-T1	Aci1	ADX94286.1
CP003850.1	SAMN02604246	BJAB0868	p1BJAB0868	8721	Rep_3	R3-T1	Aci1	AGQ12262.1
CP006964.1	SAMN03081512	AB07	pPKAB07	8805	Rep_3	R3-T1	Aci1	AHJ95281.1
CP007550.1	SAMN02471420	AC12	pAC12	8731	Rep_3	R3-T1	Aci1	AHX30527.1
CP007578.1	SAMN02471421	AC30	pAC30a	8685	Rep_3	R3-T1	Aci1	AHX67213.1
CP008708.1	SAMN02894434	AB5075-UW	p2AB5075	8731	Rep_3	R3-T1	Aci1	AKA33680.1
CP008850.1	SAMN02714232	AC29	pAC29a	8737	Rep_3	R3-T1	Aci1	AKB09309.1
CP010782.1	SAMN03248539	A1	pA1-1	8731	Rep_3	R3-T1	Aci1	AJF83584.1
CP012955.1	SAMN04029125	D36	pD36-3	9276	Rep_3	R3-T1	Aci1	ALJ89812.1
CP014217.1	SAMN04448797	YU-R612	p1YU-R612	5465	Rep_3	R3-T1	Aci1	AMC17828.1
CP014293.1	SAMN04457967	AB34299	p1AB34299	15645	Rep_3	R3-T1	Aci1	AQU58924.1
CP015486.1	SAMN03277095	ORAB01	pORAB01-3	15198	Rep_3	R3-T1	-	ANB90648.1
CP020576.1	SAMN06650263	SSA12	pSSA12_2	8730	Rep_3	R3-T1	Aci1	ARF94646.1
CP021786.1	SAMN07125723	A85	pA85-2	8731	Rep_3	R3-T1	Aci1	AHM95263.1
CP026708.1	SAMN04014897	AR_0056	tig00000534_pilon	8731	Rep_3	R3-T1	Aci1	AVE48090.1
CP027124.1	SAMN04014897	AR_0056	p1AR_0056	8731	Rep_3	R3-T1	Aci1	AVN07781.1
CP027244.1	SAMN08364584	WCHAB005078	p2_005078	8731	Rep_3	R3-T1	Aci1	AVN12717.1
CP027529.1	SAMN04014924	AR_0083	pAR_0083	8731	Rep_3	R3-T1	Aci1	AVN27928.1
CP027609.1	SAMN04014943	AR_0102	p1AR_0102	8731	Rep_3	R3-T1	-	-
CP031446.1	SAMN09769497	MDR-UNC	pAB120	10879	Rep_3	R3-T1	Aci1	QBA07847.1
CP033870.1	SAMN10411605	MRSN15313	p597A-6.7,	6667	Rep_3	R3-T1	Aci1	AYY91181.1
CP040261.1	SAMN11621520	P7774	p2P7774	14880	Rep_3	R3-T1	Aci1	QCR91174.1
CP050387.1	SAMN14409516	VB82	pVB82_2	7540	Rep_3	R3-T1	Aci1	QJH23945.1
CP050391.1	SAMN14409813	VB723	pVB723_1	8731	Rep_3	R3-T1	Aci1	QJH16202.1
CP050393.1	SAMN14410107	VB11737	pVB11737_2	8731	Rep_3	R3-T1	Aci1	QJH08912.1
CP050402.1	SAMN14410138	VB2181	pVB2181	8731	Rep_3	R3-T1	Aci1	QJH08812.1
CP050411.1	SAMN14420246	PM1912235	pPM192235_1	8731	Rep_3	R3-T1	Aci1	QJG93743.1
CP050414.1	SAMN14420253	PM192696	pPM192696_2	8732	Rep_3	R3-T1	Aci1	QJG90135.1
CP050419.1	SAMN14420254	PM193665	pPM193665_4	7540	Rep_3	R3-T1	Aci1	QJG86381.1
CP050422.1	SAMN14415334	VB2200	pVB2200_1	8731	Rep_3	R3-T1	Aci1	QJG97436.1
CP050429.1	SAMN14420255	PM194188	pPM194122_4	7549	Rep_3	R3-T1	Aci1	QJG82479.1
CP050524.1	SAMN14410111	VB7036	pVB7036_1	8730	Rep_3	R3-T1	Aci1	QJG74742.1
CP050527.1	SAMN14414747	VB2139	pVB2139_1	8731	Rep_3	R3-T1	Aci1	QJG70972.1
CP050906.1	SAMN14308892	DT-Ab057	p1DT-Ab057	8731	Rep_3	R3-T1	Aci1	QIX32739.1
CP050910.1	SAMN14308866	DT-Ab022	p1DT-Ab0	8731	Rep_3	R3-T1	Aci1	QIX36700.1
CP050913.1	SAMN14308864	DT-Ab020	p1DT-Ab020	8731	Rep_3	R3-T1	Aci1	QIX40567.1
CP050918.1	SAMN14308849	DT-Ab003	p1DT-Ab003	8731	Rep_3	R3-T1	Aci1	QIX48098.1
CP051475.1	SAMN14414778	VB2107	pVB2107_1	8731	Rep_3	R3-T1	Aci1	QJH01183.1
CP056785.1	SAMN15344688	TP1	pTP1A	8731	Rep_3	R3-T1	Aci1	QLA74147.1
CP058626.1	SAMN15437745	ATCC BAA1605	pATCCBAA1605	8731	Rep_3	R3-T1	Aci1	QLG82497.1
CP060012.1	SAMN15735522	TP2	pTP2A	8731	Rep_3	R3-T1	Aci1	QPO01560.1
CP060014.1	SAMN15738014	TP3	pTP3A	8731	Rep_3	R3-T1	Aci1	QPO05067.1
CP061526.1	SAMN12391537	CFSAN093705	pCFSAN093705	14639	Rep_3	R3-T1	-	QNV23750.1
CP066017.1	SAMN16357205	FDAARGOS_1036	pFDAARGOS_1036	10308	Rep_3	R3-T1	Aci1	QQB69061.1
CP066231.1	SAMN17073618	G20AB011	pG20AB011-2,	8731	Rep_3	R3-T1	Aci1	QQD98226.1
CP066240.1	SAMN17073615	G20AB007	pG20AB007-3	8731	Rep_3	R3-T1	Aci1	QQE02022.1
CP069842.1	SAMN16357502	FDAARGOS_1360	p1FDAARGOS_1360	14879	Rep_3	R3-T1	Aci1	QRR53505.1
CP069852.1	SAMN16357501	FDAARGOS_1359	pFDAARGOS_1359	8731	Rep_3	R3-T1	Aci1	QRR71166.1
CP071920.1	SAMN18276099	GIMC5510	pABT-897-17	13480	Rep_3	R3-T1	Aci1	QTF98049.1
CP072125.1	SAMN18396008	KSK1	p3KSK1	7540	Rep_3	R3-T1	Aci1	QTH58467.1
CP072273.1	SAMN18452234	KSK6	p3KSK6	7540	Rep_3	R3-T1	Aci1	QTK45779.1
CP072278.1	SAMN18452334	KSK7	p3KSK7	7540	Rep_3	R3-T1	Aci1	QTK62150.1
CP072283.1	SAMN18452342	KSK10	p3KSK10	7540	Rep_3	R3-T1	Aci1	QTK54004.1
CP072288.1	SAMN18452659	KSK11	p3KSK11	7540	Rep_3	R3-T1	Aci1	QTK58080.1
CP072293.1	SAMN18452698	KSK18	p3KSK18	7540	Rep_3	R3-T1	Aci1	QTK66220.1
CP072298.1	SAMN18452699	KSK19	p3KSK19	7540	Rep_3	R3-T1	Aci1	QTK70313.1
CP072303.1	SAMN18452712	KSK20	p3KSK20	7540	Rep_3	R3-T1	Aci1	QTK74379.1
CP072307.1	SAMN18452713	KSK Sensitive	p2KSKSensitive	6520	Rep_3	R3-T1	-	QTK77975.1
CP072401.1	SAMN18451305	KSK2	p3KSK2	7540	Rep_3	R3-T1	Aci1	QTL10389.1
HG380023.1	SAMN14229284	107m	ABIBUN107mP1	8731	Rep_3	R3-T1	Aci1	CDG34533.1
JACGEJ010000130.1	SAMN15501050	AbCTX19	pAbCTX19_9kb	8970	Rep_3	R3-T1	Aci1	MBL4063611.1
JHUI01000005.1	SAMN06650264	15A5	pAB5075	8819	Rep_3	R3-T1	Aci1	KGP67530.1
JX069966.1	SAMN14226317	K60	pAB120	10879	Rep_3	R3-T1	Aci1	AFO83979.1
KJ586856.1	SAMN14226822	G7	pAB-G7-1,	8731	Rep_3	R3-T1	Aci1	AHM95359.1
KR535993.1	SAMN14226627	A105	pA105-2	9830	Rep_3	R3-T1	Aci1	ALN43409.1
KU869528.1	SAMN14227234	A297(RUH875)	pA297-2	8731	Rep_3	R3-T1	Aci1	AMX23364.1
KX230793.1	SAMN14227146	MAL	pMAL-1	9810	Rep_3	R3-T1	Aci1	ANR95803.1
KY202456.1	SAMN14227361	AB1433	pIBAC_oxa58_1433	26496	Rep_3	R3-T1	Aci1	ARD69932.1
KY202457.1	SAMN14227360	AB2RED09	pIBAC_oxa58_2RED	25311	Rep_3	R3-T1	Aci1	ARD69954.1
KY202458.1	SAMN14227359	AB20C15	pIBAC_oxa58_20C15	26781	Rep_3	R3-T1	Aci1	ARD69975.1
MG954378.1	SAMN14228296	SGH0905	pS32-1	13545	Rep_3	R3-T1	Aci1	AWO68558.1
MH362811.1	SAMN07258672	11A1314CRGN088	pO237-3	18475	Rep_3	R3-T1	-	AXF37697.1
MH362812.1	SAMN07258655	11A1213CRGN008	pO237-1	15199	Rep_3	R3-T1	-	AXF37710.1
MH362813.1	SAMN07258647	11A1213CRGN055	pO237-2	18475	Rep_3	R3-T1	-	AXF37717.1
MK386683.1	SAMN14228689	ABAY14012	pABAY14012_4D	8753	Rep_3	R3-T1	Aci1	QBN23334.1
MK431775.1	SAMN14229072	11A14CRGN003	pO237-4	15199	Rep_3	R3-T1	-	QBQ02584.1
MK531537.1	-	MC1/MC23	pMC1.2/pMC23.2	8731	Rep_3	R3-T1	Aci1	QCW06040.1
CU468232.1	SAMEA3138277	SDF	p2ABSDF	25104	Rep_3	R3-T10	p2ABSDF0001	CAP02944.1

CU468232.1	SAMEA3138277	SDF	p2ABSDF	25014	Rep_3	R3-T10	p2ABSDF0001	CAP02944.1
LR026972.1	SAMEA4646212	RDK36_28	pKCRI-28-1	29606	Rep_3	R3-T10	p2ABSDF0001	-
CM003742.1	SAMN04407353	MEX11594	p1MEX11594	4437	Rep_3	R3-T10	p2ABSDF0001	-
CP051871.1	SAMN14667516	Ab-D10a-a	pAb-D10a-a_2	8495	Rep_3	R3-T10	p2ABSDF0001	QJF33703.1
CP051877.1	SAMN14667515	Ab-B004d-c	pAb-B004d-c_2	8495	Rep_3	R3-T10	p2ABSDF0001	QJF37590.1
CP059730.1	SAMN15501316	AbCTX13	pAbCTX13_7kb	7055	Rep_3	R3-T10	p2ABSDF0001	QRN23772.1
JACGEK010000079.1	SAMN15501052	AbCTX17	pAbCTX17_7kb	7055	Rep_3	R3-T10	p2ABSDF0001	MBL4076741.1
LT984691.1	SAMEA104446236	K50	p1K50	9539	Rep_3	R3-T10	p2ABSDF0001	SPC58512.1
GU978998.1	-	-	p844	1119	Rep_3	R3-T11	Aci4	ADM89093.1
CM009651.1	SAMN08093362	ZQ3	p5ZQ3	16470	Rep_3	R3-T11	Aci4	PQJ03708.1
CP040045.1	SAMN11554497	VB958	p1VB958	16485	Rep_3	R3-T11	Aci4	QCP18714.1
CP040049.1	SAMN11554995	VB1190	pVB1190	16479	Rep_3	R3-T11	Aci4	QCP22235.1
CP040055.1	SAMN11557490	VB35179	p1VB35179	16467	Rep_3	R3-T11	Aci4	QCP25971.1
CP050389.1	SAMN14409628	VB473	pVB473_1	16470	Rep_3	R3-T11	Aci4	QJH19807.1
CP050405.1	SAMN14414761	VB2486	pVB2486_2	14906	Rep_3	R3-T11	Aci4	QJH05136.1
CP050418.1	SAMN14420254	PM193665	pPM193665_3	18783	Rep_3	R3-T11	Aci4	QJG86361.1
CP050427.1	SAMN14420255	PM194188	pPM194122_2	18783	Rep_3	R3-T11	Aci4	QJG82448.1
AFDB02000003.1	SAMN00114928	Naval-81	pNaval81-26	26089	Rep_3	R3-T12	-	EJP56825.1
CM009030.2	SAMN08093369	ZQ10	p1ZQ10	35194	Rep_3	R3-T12	-	PQJ03477.1
CM009083.3	SAMN08093368	ZQ9	p1ZQ9	35194	Rep_3	R3-T12	-	PQJ03454.1
AFDA02000011.1	SAMN00114927	Naval-18	pNaval18-7.0	7032	Rep_3	R3-T12	-	EJP48327.1
ALII01000018.1	SAMN02436485	IS-123	pIS123-12	11600	Rep_3	R3-T12	-	EJO37651.1
CP045108.1	SAMN12389466	ATCC 19606	p1ATCC19606	7655	Rep_3	R3-T12	-	QFQ03441.1
CP065888.1	SAMN13450447	FDAARGOS_917	p1FDAARGOS_917	6778	Rep_3	R3-T12	-	QQA28272.1
AFDA02000008.1	SAMN00114927	Naval-18	pNaval18-8.4	8422	Rep_3	R3-T13	-	EJP48482.1
CP010400.1	SAMN03263969	6200	p6200-9.327kb	9327	Rep_3	R3-T13	-	AJB69037.1
CP026126.1	SAMN06040401	ABNIH28	pABA-6973	11305	Rep_3	R3-T13	-	AUT39979.1
CP044521.1	SAMN12859885	29FS20	p29FS20-2	12731	Rep_3	R3-T13	-	QLF12502.1
CP051865.1	SAMN14667518	Ab-C102	pAb-C102_3	19853	Rep_3	R3-T13	-	QJF29816.1
CP059388.1	SAMN15541804	36-1512	p2.36-1512	9458	Rep_3	R3-T13	-	QLY88340.1
CP033871.1	SAMN10411605	MRSN15313	p597A-14.8	14087	Rep_3	R3-T14	-	AYY91191.1
MG100202.1	SAMN14228542	Ab825	pAb825_36	35743	Rep_3	R3-T14	-	AVR61203.1
MN266872.1	-	N/A	pAC1-BRL	16673	Rep_3	R3-T14	-	QHW11287.1
KY984045.1	SAMN07509424	AB242	pAb242_9	9284	Rep_3	R3-T14	-	AUO31880.1
MG520098.1	SAMN14228448	Ab244	pAb244_7	7965	Rep_3	R3-T14	-	AVX50861.1
MK323042.1	SAMN14228622	Acb-45063	pAb45063_a	19808	Rep_3	R3-T14	-	QBK17989.1
MK531541.1	-	MC75	pMC75.2	13903	Rep_3	R3-T14	-	QCO89772.1
CP062923.1	SAMN16304032	Res13-Abat-PEA21-P4-01-A	p1Res13-Abat	5242	Rep_3	R3-T15	-	QPF15413.1
AFDL01000005.1	SAMN02436631	OIFC143	pOIFC143-2.3	2277	Rep_3	R3-T15	-	EJG16531.1
CM009646.1	SAMN08093369	ZQ10	p3ZQ10	2277	Rep_3	R3-T15	-	PQJ03470.1
JADAIY010000264.1	SAMN16304042	Res13-Abat-PEA28-P5-02-A	pRes13-Abat-PEA28-P5-02-A	2279	Rep_3	R3-T15	-	-
MK386684.1	SAMN14228688	ABAY15001	pABAY15001_6E	2278	Rep_3	R3-T15	-	QBN23345.1
CM016516.1	SAMN10261616	TG31307	pTG31307	55269	Rep_3	R3-T16	-	THJ58480.1
CP039342.1	SAMN10261613	TG31986	pTG31986	55269	Rep_3	R3-T16	-	QCD20890.1
CP039344.1	SAMN10261535	TG31302	pTG31302	55268	Rep_3	R3-T16	-	QCD24650.1
CP039932.1	SAMN10261537	TG29392	pTG29392_2	55269	Rep_3	R3-T16	-	QCO80749.1
CP072306.1	SAMN18452713	KSK Sensitive	p1KSKSensitive	55356	Rep_3	R3-T16	-	QTK77957.1
CP015365.1	SAMN04485290	3207	pAba3207a	13478	Rep_3	R3-T17	-	ANC38759.1
CP022284.1	SAMN07289440	7804	pAba7804a	12381	Rep_3	R3-T17	-	ASO73059.1
CP023032.1	SAMN07284119	7847	pAba7847a	13478	Rep_3	R3-T17	-	AXW92573.1
CP033244.1	SAMN07520235	7835	pAba7835a	8536	Rep_3	R3-T17	-	QFY70909.1
CP050428.1	SAMN14420255	PM194188	pPM194122_3	7695	Rep_3	R3-T17	-	QJG82477.1
CU459137.1	SAMEA3138279	AYE	p1ABAYE	5644	Rep_3	R3-T18	p1ABAYE0001	CAM84608.1
CU459137.1	SAMEA3138279	AYE	p1ABAYE	5644	Rep_3	R3-T18	p1ABAYE0001	CAM84608.1
CM004453.1	SAMN03699770	M3AC14-8	p1M3AC14-8	5441	Rep_3	R3-T18	p1ABAYE0001	OBR16604.1
CP042561.1	SAMN12289292	E47	pE47_005	5234	Rep_3	R3-T18	p1ABAYE0001	QFH47707.1
CP046901.1	SAMN13565236	A1429	pA1429a	7852	Rep_3	R3-T18	p1ABAYE0001	QLB37634.1
CP024419.1	SAMN07736509	A388	pA388	33036	Rep_3	R3-T19	Aci10	ATP89028.1
CP031381.2	SAMN09302593	ACICU	pACICU1b	24268	Rep_3	R3-T19	Aci10	QCS03997.1
CP027179.1	SAMN04014911	AR_0070	p2AR_0070	39535	Rep_3	R3-T19	Aci10	AVI35083.1
CP027184.1	SAMN04014893	AR_0052	p3AR_0052	60699	Rep_3	R3-T19	Aci10	AVI39286.1
FM210331.1	SAMN14229501	VA-566/00	pABVA01	8963	Rep_3	R3-T2	Aci2	CAR65318.1
FM210331.1	SAMN14229501	VA-566/00	pABVA01	8963	Rep_3	R3-T2	Aci2	CAR65318.1
MN495625.1	-	A2485	pA2485	15405	Rep_3	R3-T2	Aci2	QID24189.1
MN495626.1	-	A2503	pA2503	15405	Rep_3	R3-T2	Aci2	QID24208.1
AP022240.1	SAMD00194616	WP8-W18-ESBL-11	pWP8-W18-ESBL-11_2	10735	Rep_3	R3-T2	-	-
AYFI01000019.1	SAMN07736509	A388	pABUH6b-10	10030	Rep_3	R3-T2	Aci2	ETR11343.1
CM009045.2	SAMN08093363	ZQ4	p1ZQ4	8331	Rep_3	R3-T2	Aci2	PST50036.1
CP026748.1	SAMN08364585	WCHAB005133	p1_005133	5602	Rep_3	R3-T2	Aci2	AVE88593.1
CP029573.1	SAMN06650239	HWBA8	pDA33098-9	8963	Rep_3	R3-T2	Aci2	AWO18631.1
CP035044.1	SAMN05238571	ABUH796	p13.0Kbp	12952	Rep_3	R3-T2	Aci2	QAS95996.1
CP035048.1	SAMN05238672	ABUH793	p10.9Kbp	10945	Rep_3	R3-T2	Aci2	QAS99819.1
CP035050.1	SAMN05238697	ABUH773	p11.8Kbp	11810	Rep_3	R3-T2	Aci2	QAT03382.1
CP035053.1	SAMN05238628	YU-R612	p11.0Kbp	10967	Rep_3	R3-T2	Aci2	QAT07114.1
CP039521.1	SAMN10261584	TG22627	pTG22627	5602	Rep_3	R3-T2	Aci2	QCH38679.1
CP039995.1	SAMN10261544	TG22182	pTG22182_2	5602	Rep_3	R3-T2	Aci2	QCO84567.1
CP040427.1	SAMN11660471	PB364	pPB364_2	10967	Rep_3	R3-T2	Aci2	QCT18126.1
CP041589.1	SAMN12158047	J9	pJ9-2	10967	Rep_3	R3-T2	Aci2	QDM68406.1
CP042208.1	SAMN08637743	DS002	pTS11291	11291	Rep_3	R3-T2	Aci2	QDX16375.1
CP042560.1	SAMN12289292	E47	pE47_004	7703	Rep_3	R3-T2	Aci2	QFH47694.1
CP044358.1	SAMN12825295	CAM180-1	pCAM180B	16096	Rep_3	R3-T2	Aci2	QEY06144.1
CP047976.1	SAMN13884837	DETAB-P2	pDETAB3	9132	Rep_3	R3-T2	Aci2	QMS84177.1
CP050434.1	SAMN14422682	PM194229	pPM194229_2	10697	Rep_3	R3-T2	Aci2	QJG78557.1
CP059389.1	SAMN15541804	36-1512	p3.36-1512	5688	Rep_3	R3-T2	Aci2	QLY88346.1
CP067103.1	SAMN12399660	ATCC BAA-1790	pNC2	10955	Rep_3	R3-T2	Aci2	QQY87247.1
GQ904227.1	SAMN14225470	-	pMMCU3	8964	Rep_3	R3-T2	Aci2	ADB23472.1
HG977525.1	SAMEA3158456	CS01	pCS01C	8174	Rep_3	R3-T2	Aci2	-
HG977529.1	SAMEA3158506	CR17	pCR17C	8047	Rep_3	R3-T2	Aci2	-
KJ534568.1	SAMN14226773	ATCC 223	AbATCC223	8840	Rep_3	R3-T2	Aci2	AIA61624.1
KJ534569.1	SAMN14226772	ATCC 329	AbATCC329	8842	Rep_3	R3-T2	Aci2	AIA61634.1
KM051986.1	SAMN14226464	D72	pD72-1	10967	Rep_3	R3-T2	Aci2	AIH07953.1
MG954376.1	SAMN14228298	SGH9601	pS21-1	12952	Rep_3	R3-T2	Aci2	AWO68412.1
CP051870.1	SAMN14667516	Ab-D10a-a	pAb-D10a-a_1	48239	Rep_3	R3-T20	-	QJF33663.1
CP051876.1	SAMN14667515	Ab-B004d-c	pAb-B004d-c_1	48239	Rep_3	R3-T20	-	QJF37552.1
CP053220.1	SAMN14833556	DT01139C	p2DT01139C	63650	Rep_3	R3-T20	-	QLI41749.1
KY216144.1	-	RCH51	pRCH51-3	52789	Rep_3	R3-T20	-	AQT19035.1
CP026129.1	SAMN06040401	ABNIH28	pABA-2f10	130044	Rep_3	R3-T21	-	AUT40247.1
CP026749.2	SAMN08364585	WCHAB005133	pOXA58_005133,	42455	Rep_3	R3-T21	-	AVE88635.1
CP038501.1	SAMN11298775	CIAT758	p3CIAT758	78125	Rep_3	R3-T21	-	QBY16306.1
CP053219.1	SAMN14833556	DT01139C	p3DT01139C	97161	Rep_3	R3-T21	-	QLI41705.1
CM013137.1	SAMN10662617	AB18PR065	pAB18PR065-MCR-4.3	25602	Rep_3	R3-T22	-	RUT37677.1
CP033872.1	SAMN10411605	MRSN15313	pAb-MCR4.1	35502	Rep_3	R3-T22	-	AYY91210.1

CP038261.1	SAMN10386508	39741	pEH_mcr4.3	18786	Rep_3	R3-T22	-	QBR82861.1
CP038265.1	SAMN10386510	LEV1449/17Ec	pEC_mcr4.3	43093	Rep_3	R3-T22	-	QBR79279.1
AFDB02000005.1	SAMN00114928	Naval-81	pNaval81-13	12634	Rep_3	R3-T23	-	-
AFDO01000021.1	SAMN02436551	Naval-17	pNaval17-13	12636	Rep_3	R3-T23	-	EJG28245.1
CM009033.2	SAMN08093367	ZQ8	p1ZQ8	12636	Rep_3	R3-T23	-	PQL85636.1
CP050406.1	SAMN14414761	VB2486	pVB2486_3	12574	Rep_3	R3-T23	-	QJH05158.1
CP033871.1	SAMN10411605	MRSN15313	p597A-14.8	14087	Rep_3	R3-T24	-	AYY91202.1
CP034097.1	SAMN10441121	A52	pA52-OXA-72	8493	Rep_3	R3-T24	-	QAB42528.1
CP043954.1	SAMN12769618	K09-14	pK09-14	7791	Rep_3	R3-T24	-	QER77251.1
KY704308.1	SAMN14227859	IHIT32296	pAbIHIT32296	8493	Rep_3	R3-T24	-	ASN73624.1
CP012956.1	SAMN04029125	D36	pD36-4	47457	Rep_3	R3-T25	-	ALJ89824.1
CP051863.1	SAMN14667518	Ab-C102	pAb-C102_1	90089	Rep_3	R3-T25	-	QJF29769.1
CP051867.1	SAMN14667517	Ab-C63	pAb-C63_1	81353	Rep_3	R3-T25	-	QJF41224.1
AYFH01000057.1	SAMN02597386	UH7607	pABUH2b-5.4	5355	Rep_3	R3-T26	-	ETR11099.1
CP026340.1	SAMN07559626	810CP	pAba810CPa	5281	Rep_3	R3-T26	-	AXG87071.1
CP044518.1	SAMN12860376	31FS3-2	p31FS3-2-1	6099	Rep_3	R3-T26	-	QLF08691.1
CP034094.1	SAMN10441121	A52	pA52-2	27452	Rep_3	R3-T27	-	QAB42494.1
CP051864.1	SAMN14667518	Ab-C102	pAb-C102_2	67097	Rep_3	R3-T27	-	QJF29792.1
CP059478.1	SAMN15637465	17-84	p17-84_OXA	108715	Rep_3	R3-T28	-	QNB01597.1
CP047975.1	SAMN13884837	DETAB-P2	pDETAB2	100072	Rep_3	R3-T28	-	QMS84089.1
LN833432.1	SAMEA3298506	CHI-32	pNDM-32	84623	Rep_3	R3-T28	-	-
CP062922.1	SAMN16304032	Res13-Abat-PEA21-P4-01-A	p2Res13-Abat	14288	Rep_3	R3-T29	-	QPF15404.1
JADAJJ010000299.1	SAMN16304029	Res13-Abat-PEA16-P5-01-A	pRes13-Abat-PEA16-P5-01-A	14288	Rep_3	R3-T29	-	-
JADAJL010000301.1	SAMN16304027	Res13-Abat-EA3-S5-02-A	pRes13-Abat-EA3-S5-02-A	14288	Rep_3	R3-T29	-	-
AFCZ02000003.1	SAMN00114924	OIFC032	pOIFC032-101	101298	Rep_3	R3-T3	-	EJP40261.1
AFDM01000010.1	SAMN00114925	OIFC189	pOIFC189-111	110967	Rep_3	R3-T3	-	EJG16262.1
AP022239.1	SAMD00194616	WP8-W18-ESBL-11	pWP8-W18-ESBL-11_1	114365	Rep_3	R3-T3	-	-
AYFW01000101.1	SAMN02597401	UH2107	pABUH4-111	111007	Rep_3	R3-T3	-	ETS68256.1
CM008330.1	SAMN04272870	AC002-1-R4	pAC0021R4	111165	Rep_3	R3-T3	-	PCO03588.1
CM009049.2	SAMN08093361	ZQ2	p1ZQ2	110967	Rep_3	R3-T3	-	PQJ03931.1
CP004359.1	SAMN02603104	MDR-TJ	pABTJ2	110967	Rep_3	R3-T3	-	AGG91013.1
CP006769.1	SAMN02641530	ZW85-1	ZW85p2	113866	Rep_3	R3-T3	-	AHB93304.1
CP010398.1	SAMN03263969	6200	p6200-114.848kb	114848	Rep_3	R3-T3	-	AJB68963.1
CP010780.1	SAMN03290686	XH386	pAB386	112157	Rep_3	R3-T3	-	AKJ47876.1
CP015484.1	SAMN03277095	ORAB01	pORAB01-1	110965	Rep_3	R3-T3	-	ANB90502.1
CP016296.1	SAMN04096368	CMC-CR-MDR-Ab4	pCMCVTAb1-Ab4	110968	Rep_3	R3-T3	-	APQ87295.1
CP016299.1	SAMN04096369	CMC-MDR-Ab59	pCMCVTAb1-Ab59	110967	Rep_3	R3-T3	-	APQ91163.1
CP016301.1	SAMN04096370	CMC-CR-MDR-Ab66	pCMCVTAb1-Ab66	110967	Rep_3	R3-T3	-	APQ94950.1
CP018257.1	SAMN06077192	AF-673	pAF-673	110964	Rep_3	R3-T3	-	APJ25221.1
CP018333.1	SAMN06099024	A1296	pA1296_1	112773	Rep_3	R3-T3	-	ATI40464.1
CP020585.1	SAMN06650245	CBA7	pCBA7_1	111999	Rep_3	R3-T3	-	ARG11462.1
CP021327.1	SAMN07135565	XH386	pXH386	112155	Rep_3	R3-T3	-	AWW83408.1
CP023025.1	SAMN07520237	10324	pAba10324c	113420	Rep_3	R3-T3	-	AXX47015.1
CP024125.1	SAMN07445112	AYP-A2	pAYP-A2	110967	Rep_3	R3-T3	-	ATU25204.1
CP026128.1	SAMN06040401	ABNIH28	pABA-1fe1	110754	Rep_3	R3-T3	-	AUT40134.1
CP026945.1	SAMN07977762	S1	pAbS1_02	111068	Rep_3	R3-T3	-	AVG28499.1
CP027182.1	SAMN04014911	AR_0070	p4AR_0070	105127	Rep_3	R3-T3	-	AVI35343.1
CP027185.1	SAMN04014893	AR_0052	p4AR_0052	105128	Rep_3	R3-T3	-	AVI39456.1
CP027483.1	SAMN07689236	I43	pABI43	114918	Rep_3	R3-T3	-	AVN23945.1
CP028139.1	SAMN08513260	NCIMB 8209	pAbNCIMB8209_134	133709	Rep_3	R3-T3	-	QBC49395.1
CP032217.1	SAMN09906510	UPAB1	p2UPAB1	80061	Rep_3	R3-T3	-	-
CP034093.1	SAMN10441121	A52	pA52-1	110713	Rep_3	R3-T3	-	QAB42408.1
CP036284.1	SAMN10261590	TG60155	p60155_1	127784	Rep_3	R3-T3	-	QBH55712.1
CP038645.1	SAMN11311113	ACN21	p8ACN21	116047	Rep_3	R3-T3	-	QBY91555.1
CP039519.1	SAMN10261589	TG22653	pTG22653	127784	Rep_3	R3-T3	-	QCH35063.1
CP041590.1	SAMN12158047	J9	pJ9-3	145071	Rep_3	R3-T3	-	QDM68418.1
CP046537.1	SAMN13476302	XL380	pXL380	112007	Rep_3	R3-T3	-	QGW12467.1
CP047974.1	SAMN13884837	DETAB-P2	pDETAB1	103751	Rep_3	R3-T3	-	QMS84036.1
CP050905.1	SAMN14308892	DT-Ab057	p2DT-Ab057	110967	Rep_3	R3-T3	-	QIX32630.1
CP050908.1	SAMN14308866	DT-Ab022	p3DT-Ab022	119067	Rep_3	R3-T3	-	QIX36501.1
CP059387.1	SAMN15541804	36-1512	p1.36-1512	113330	Rep_3	R3-T3	-	QLY88253.1
CP061515.1	SAMN12391854	CFSAN093710	pCFSAN093710_1	110967	Rep_3	R3-T3	-	QNV19832.1
CP061518.1	SAMN12413929	CFSAN093709	pCFSAN093709	110965	Rep_3	R3-T3	-	QNV16036.1
CP061520.1	SAMN12391536	CFSAN093708	pCFSAN093708	110967	Rep_3	R3-T3	-	QNV35117.1
CP061522.1	SAMN12391534	CFSAN093707	pCFSAN093707	110964	Rep_3	R3-T3	-	QNV31204.1
CP061524.1	SAMN12391535	CFSAN093706	pCFSAN093706	110967	Rep_3	R3-T3	-	QNV27490.1
CP062920.1	SAMN16304032	Res13-Abat-PEA21-P4-01-A	p4Res13-Abat	113139	Rep_3	R3-T3	-	QPF15204.1
CP072527.1	SAMN18498586	DETAB-E227	pDETAB4	113682	Rep_3	R3-T3	-	QTM22032.1
MK386680.1	SAMN14228692	ABAY04001	pABAY04001_1A	110967	Rep_3	R3-T3	-	QBN23070.1
CP050420.1	SAMN14420254	PM193665	pPM193665_5	2762	Rep_3	R3-T30	-	QJG86394.1
CP050430.1	SAMN14420255	PM194188	pPM194122_5	2762	Rep_3	R3-T30	-	QJG82492.1
GU978999.1	-	-	p537	1125	Rep_3	R3-T31	Aci5	ADM89094.1
LN865144.1	SAMEA3449716	CIP70.10	pCIP70.10	7742	Rep_3	R3-T31	Aci5	CRL96367.1
LN997847.1	SAMEA3715145	R2091	pR2091	7742	Rep_3	R3-T31	Aci5	CUW37058.1
GU979000.1	-	-	p11921	1103	Rep_3	R3-T32	Aci8	ADM89095.1
KY984047.1	SAMN07509424	AB242	pAb242_25	24808	Rep_3	R3-T32	Aci8	AUO31913.1
MG100202.1	SAMN14228542	Ab825	pAb825_36	35743	Rep_3	R3-T32	Aci8	AVR61183.1
CP033751.1	SAMN10163233	FDAARGOS_540	p1FDAARGOS_540	7181	Rep_3	R3-T33	-	AYX85221.1
LR026974.1	SAMEA4646219	RDK39_49	pKCRI-49-1	11681	Rep_3	R3-T33	-	-
CP018334.1	SAMN06099024	A1296	pA1296_2	11586	Rep_3	R3-T34	-	ATI40537.1
CP059304.1	SAMN15574350	AC1633	pAC1633-3	9950	Rep_3	R3-T34	-	QOJ62416.1
AFDB02000003.1	SAMN00114928	Naval-81	pNaval81-26	26089	Rep_3	R3-T35	-	EJP56831.1
ALII01000019.1	SAMN02436485	IS-123	pIS123-18	17984	Rep_3	R3-T35	-	EJO37647.1
KY984047.1	SAMN07509424	AB242	pAb242_25	24808	Rep_3	R3-T36	-	AUO31910.1
MG100202.1	SAMN14228542	Ab825	pAb825_36	35743	Rep_3	R3-T36	-	AVR61186.1
CP000522.1	SAMN02604331	ATCC 17978	pAB1	13408	Rep_3	R3-T37	A1S_3471	ABO13850.1
CP000522.1	SAMN02604331	ATCC 17978	pAB1	13408	Rep_3	R3-T37	A1S_3471	ABO13860.1
CU468231.1	SAMEA3138277	SDF	p1ABSDF	6106	Rep_3	R3-T38	p1ABSDF0001	CAP02936.1
CU468231.1	SAMEA3138277	SDF	p1ABSDF	6106	Rep_3	R3-T38	p1ABSDF0001	CAP02936.1
CU468232.1	SAMEA3138277	SDF	p2ABSDF	25104	Rep_3	R3-T39	p2ABSDF0025	CAP02966.1
CU468232.1	SAMEA3138277	SDF	p2ABSDF	25014	Rep_3	R3-T39	p2ABSDF0025	CAP02966.1
AY541809.1	SAMN14224286	19606	pMAC	9540	Rep_3	R3-T4	Aci9	AAT09649.1
CM009036.2	SAMN08093366	ZQ7	p1ZQ7	11191	Rep_3	R3-T4	Aci9	PST49955.1
CM009653.1	SAMN08093365	ZQ6	p5ZQ6	16476	Rep_3	R3-T4	-	PQL72257.1
CM012225.1	SAMN07815360	PIMB13AB-41	pAB13-41	11194	Rep_3	R3-T4	Aci9	-
CP015122.1	SAMN04621185	ab736	pab736	9539	Rep_3	R3-T4	Aci9	ARN32819.1
CP024577.1	SAMN07945345	AbPK1	pAbPK1a	15113	Rep_3	R3-T4	-	ATR89551.1
CP027180.1	SAMN04014911	AR_0070	p3AR_0070	73018	Rep_3	R3-T4	Aci9	AVI35133.1
CP027186.1	SAMN04014893	AR_0052	p2AR_0052	38505	Rep_3	R3-T4	Aci9	AVI39478.1
CP035673.1	SAMN07977426	VB23193	pVB23193	16033	Rep_3	R3-T4	Aci9	QBB78281.1
CP035933.1	SAMN10170272	VB31459	p1VB31459	11195	Rep_3	R3-T4	Aci9	QBF38536.1
CP040086.1	SAMN11571816	VB33071	p1VB33071	11194	Rep_3	R3-T4	Aci9	QCP44014.1

CP040088.1	SAMN11571817	VB35575	pVB35575	11194	Rep_3	R3-T4	Aci9	QCP47688.1
CP045109.1	SAMN12389466	ATCC 19606	p2ATCC19606	9540	Rep_3	R3-T4	Aci9	QFQ03455.1
CP065433.1	SAMN08687991	ATCC 17961	pAB17961-1	9395	Rep_3	R3-T4	Aci9	QPP16179.1
CP065886.1	SAMN13450447	FDAARGOS_917	p2FDAARGOS_917	9540	Rep_3	R3-T4	Aci9	QQA24590.1
LT594096.1	SAMEA2439285	BAL062	pBAL062	8015	Rep_3	R3-T4	Aci9	SBS23985.1
CU468233.1	SAMEA3138277	SDF	p3ABSDF	24922	Rep_3	R3-T40	p3ABSDF0002	CAP02976.1
CU468233.1	SAMEA3138277	SDF	p3ABSDF	24922	Rep_3	R3-T40	p3ABSDF0002	CAP02976.1
CU468233.1	SAMEA3138277	SDF	p3ABSDF	24922	Rep_3	R3-T41	p3ABSDF0009	CAP02983.1
CU468233.1	SAMEA3138277	SDF	p3ABSDF	24922	Rep_3	R3-T41	p3ABSDF0009	CAP02983.1
CU468233.1	SAMEA3138277	SDF	p3ABSDF	24922	Rep_3	R3-T42	p3ABSDF0018	CAP02992.1
CU468233.1	SAMEA3138277	SDF	p3ABSDF	24922	Rep_3	R3-T42	p3ABSDF0018	CAP02992.1
GU978996.1	-	-	p736	1065	Rep_3	R3-T43	Aci7	ADM89091.1
KT346360.1	SAMN14226727	RCH52	pRCH52-1	11164	Rep_3	R3-T43	-	ALC76579.1
CP033753.1	SAMN10163233	FDAARGOS_540	p3FDAARGOS_540	86551	Rep_3	R3-T44	-	AYX85290.1
CP044520.1	SAMN12859885	29FS20	p29FS20-1	66277	Rep_3	R3-T45	-	QLF12435.1
CP038259.1	SAMN10386508	39741	pEH_gr13	135229	Rep_3	R3-T46	-	QBR82727.1
CP042565.1	SAMN12289292	E47	pE47_009	2427	Rep_3	R3-T47	-	-
CP062923.1	SAMN16304032	Res13-Abat-PEA21-P4-01-A	p1Res13-Abat	5242	Rep_3	R3-T48	-	QPF15412.1
AYFZ01000080.2	SAMN02597404	UH19608	pABUH2a-5.6	5636	Rep_3	R3-T49	-	ETQ55253.2
GU978997.1	-	-	p203	1068	Rep_3	R3-T5	Aci3	ADM89092.1
CP021348.1	SAMN03771402	B8300	pB8300	25150	Rep_3	R3-T5	-	KMV24627.1
CP038260.1	SAMN10386508	39741	pEH_gr3	25856	Rep_3	R3-T5	-	QBR82828.1
LR026972.1	SAMEA4646212	RDK36_28	pKCRI-28-1	29606	Rep_3	R3-T5	Aci3	-
AFDL01000007.1	SAMN04014897	AR_0056	pOIFC143-6.2	6241	Rep_3	R3-T5	Aci3	EJG16427.1
CP003968.1	SAMN02603576	D1279779	pD1279779	7416	Rep_3	R3-T5	Aci3	AGH37280.1
CP007713.1	SAMN02709859	LAC-4	pABLAC1	8006	Rep_3	R3-T5	Aci3	AIY39145.1
CP018255.1	SAMN06077191	AF-401	pAF-401	17583	Rep_3	R3-T5	Aci3	APJ21535.1
CP018678.1	SAMN05362953	LAC4	pALAC4-1	8006	Rep_3	R3-T5	Aci3	APO60695.1
CP023024.1	SAMN07520237	10324	pAba10324b	7143	Rep_3	R3-T5	Aci3	AXX46908.1
CP040083.1	SAMN11571814	SP304	pSP304	9185	Rep_3	R3-T5	Aci3	QCP40396.1
CP050435.1	SAMN14422682	PM194229	pPM194229_3	9847	Rep_3	R3-T5	-	QJG78569.1
CP065434.1	SAMN08687991	ATCC 17961	pAB17961-2	6667	Rep_3	R3-T5	-	QPP16194.1
CP072529.1	SAMN18498586	DETAB-E227	pDETAB6	7145	Rep_3	R3-T5	Aci3	QTM22127.1
CP046900.1	SAMN13565236	A1429	pA1429b	19147	Rep_3	R3-T50	-	QLB37617.1
CM009050.2	SAMN08093361	ZQ2	p4ZQ2	5695	Rep_3	R3-T51	-	PQJ03811.1
CP053216.1	SAMN14833494	DT0544C	p2DT0544C	55394	Rep_3	R3-T52	-	QLI38221.1
CP030107.1	SAMN09460321	DA33382	pDA33382-2-2	2372	Rep_3	R3-T53	-	AXB17573.1
CP040262.1	SAMN11621520	P7774	p1P7774	5464	Rep_3	R3-T54	-	QCR91187.1
CP042564.1	SAMN12289292	E47	pE47_008	3065	Rep_3	R3-T55	-	QFH47722.1
CP050408.1	SAMN14414761	VB2486	pVB2486_5	5432	Rep_3	R3-T56	-	QJH05174.1
CP023023.1	SAMN07520237	10324	pAba10324a	5300	Rep_3	R3-T57	-	AXX46897.1
CP034096.1	SAMN10441121	A52	pA52-4	3610	Rep_3	R3-T58	-	QAB42527.1
AP023080.1	SAMD00059694	OCU_Ac16a	pOCU_Ac16a_3	13096	Rep_3	R3-T59	-	-
CM009030.2	SAMN08093369	ZQ10	p1ZQ10	35194	Rep_3	R3-T6	-	PQJ03509.1
CM009083.3	SAMN08093368	ZQ9	p1ZQ9	35194	Rep_3	R3-T6	-	PQJ03469.1
CP021348.1	SAMN03771402	B8300	pB8300	25150	Rep_3	R3-T6	-	KMV24615.1
CP038260.1	SAMN10386508	39741	pEH_gr3	25856	Rep_3	R3-T6	-	QBR82859.1
CP059478.1	SAMN15637465	17-84	p17-84_OXA	108715	Rep_3	R3-T6	-	QNB01606.1
AYFH01000048.1	SAMN02597386	UH7607	pABUH3a-8.2	8190	Rep_3	R3-T6	-	ETR11568.1
AYFZ01000083.1	SAMN02597404	UH19608	pABUH3b-7.8	7819	Rep_3	R3-T6	-	ETQ55058.1
CM009035.2	SAMN08093367	ZQ8	p3ZQ8	11034	Rep_3	R3-T6	-	PQL85655.1
CM009046.2	SAMN08093363	ZQ4	p2ZQ4	13311	Rep_3	R3-T6	-	PST50007.1
CP033752.1	SAMN10163233	FDAARGOS_540	p2FDAARGOS_540	13195	Rep_3	R3-T6	-	AYX85239.1
CP038648.1	SAMN11311113	ACN21	p5ACN21	9205	Rep_3	R3-T6	-	QBY91635.1
CP042207.1	SAMN08637743	DS002	pTS9900	9900	Rep_3	R3-T6	-	QDX16364.1
CP051868.1	SAMN14667517	Ab-C63	pAb-C63_2	10663	Rep_3	R3-T6	-	QJF41245.1
CM009647.1	SAMN08093361	ZQ2	p2ZQ2	12769	Rep_3	R3-T60		PQJ03830.1
CP059302.1	SAMN15574350	AC1633	pAC1633-4	5210	Rep_3	R3-T61	-	QOJ62393.1
KY617771.1	SAMN14227876	SGH0823	pS30-1	18234	Rep_3	R3-T62	-	ARH59503.1
CP012956.1	SAMN04029125	D36	pD36-4	47457	Rep_3	R3-T63	-	ALJ89842.1
CP053221.1	SAMN14833556	DT01139C	p1DT01139C	9613	Rep_3	R3-T64	-	QLI41769.1
LR026973.1	SAMEA4646218	RDK37_43	pKCRI-43-1	34935	Rep_3	R3-T65	-	-
CP042559.1	SAMN12289292	E47	pE47_003	8795	Rep_3	R3-T66	-	QFH47692.1
CP059303.1	SAMN15574350	AC1633	pAC1633-2	12651	Rep_3	R3-T67	-	QOJ62400.1
KY984046.1	SAMN07509424	AB242	pAb242_12	11891	Rep_3	R3-T68	-	AUO31881.1
GQ861437.1	-	-	135040	3975	Rep_3	R3-T69	rep135040	ACX70400.1
CU459140.1	SAMEA3138279	AYE	p3ABAYE	94413	Rep_3	R3-T7	p3ABAYE0002	CAM84695.1
CU459140.1	SAMEA3138279	AYE	p3ABAYE	94413	Rep_3	R3-T7	p3ABAYE0002	CAM84695.1
CP027245.2	SAMN08364584	WCHAB005078	pOXA58_005078	70509	Rep_3	R3-T7	-	AVN12752.1
CP033769.1	SAMN10163228	FDAARGOS_533	p2FDAARGOS_533	97783	Rep_3	R3-T7	p3ABAYE0002	AYY55194.1
CP038263.1	SAMN10386510	LEV1449/17Ec	pEC_gr13	128013	Rep_3	R3-T7	p3ABAYE0002	QBR79086.1
CP041149.1	SAMN12057668	CUVET-MIC596	pCUVET596	82016	Rep_3	R3-T7	-	QJP32841.1
CP042210.1	SAMN08637743	DS002	pTS134338	134338	Rep_3	R3-T7	p3ABAYE0002	-
CP042558.1	SAMN12289292	E47	pE47_002	59744	Rep_3	R3-T7	p3ABAYE0002	QFH47659.1
CP044357.1	SAMN12825295	CAM180-1	pCAM180A	92034	Rep_3	R3-T7	p3ABAYE0002	QEY06053.1
CP072528.1	SAMN18498586	DETAB-E227	pDETAB5	97035	Rep_3	R3-T7	-	QTM22087.1
KT852971.1	SAMN14226423	255_n	p255n_1	92939	Rep_3	R3-T7	-	AMD83595.1
AY228470.1	-	-	pAB02	4162	Rep_3	R3-T8	repA_ABE	AAR00517.1
EU294228.1	SAMN14225727	nk	pABIR	29823	Rep_3	R3-T8	RepA_AB	ACB05788.1
MN495625.1	-	A2485	pA2485	15405	Rep_3	R3-T8	repA_ABE	QID24195.1
MN495626.1	-	A2503	pA2503	15405	Rep_3	R3-T8	repA_ABE	QID24215.1
CP023021.1	SAMN07520236	9201	pAba9201a	9024	Rep_3	R3-T8	repA_ABE	AXX43430.1
CP023027.1	SAMN07520233	10042	pAba10042a	10062	Rep_3	R3-T8	repA_ABE	AXX50757.1
CP023035.1	SAMN07520231	5845	pAba5845a	9935	Rep_3	R3-T8	repA_ABE	AXX58329.1
CP029572.1	SAMN09241862	DA33098	pDA33098-9-2	8771	Rep_3	R3-T8	repA_ABE	AWO18623.1
GQ476987.1	SAMN14225384	CU2	pMMCU2	10270	Rep_3	R3-T8	repA_ABE	ACY68271.1
GQ904226.1	SAMN14225471	-	pMMD	9964	Rep_3	R3-T8	repA_ABE	ADB23463.1
KT022421.1	SAMN14226656	ML	pAB-ML	12056	Rep_3	R3-T8	repA_ABE	-
AYOI01000002.1	SAMN02597423	UH10707	pABUH5-114	114115	Rep_3	R3-T9	-	ETR59191.1
CP026705.1	SAMN04014897	AR_0056	tig00000058_pilon	113706	Rep_3	R3-T9	-	AVE44280.1
CP026944.1	SAMN07977762	S1	pAbS1_01	108394	Rep_3	R3-T9	-	AVG28377.1
CP027122.1	SAMN04014897	AR_0056	p3AR_0056	113706	Rep_3	R3-T9	-	AVN03837.1
CP027608.1	SAMN04014943	AR_0102	p2AR_0102	106967	Rep_3	R3-T9	-	-
CP029570.1	SAMN09241862	DA33098	pDA33098-108	108151	Rep_3	R3-T9	-	AWO18398.1
CP031445.1	SAMN09769497	MDR-UNC	pMDR-UNC	112216	Rep_3	R3-T9	-	QBA07831.1
CP035046.1	SAMN05238672	ABUH793	p107.0Kbp	106963	Rep_3	R3-T9	-	QAS99596.1
CP040426.1	SAMN11660471	PB364	pPB364_1	111449	Rep_3	R3-T9	-	QCT18072.1
